# Selection and phenotypic characterization of a core collection of *Brachypodium distachyon* inbred lines

**DOI:** 10.1186/1471-2229-14-25

**Published:** 2014-01-14

**Authors:** Ludmila Tyler, Jonatan U Fangel, Alexandra Dotson Fagerström, Michael A Steinwand, Theodore K Raab, William GT Willats, John P Vogel

**Affiliations:** 1 USDA-ARS Western Regional Research Center, Albany, CA, USA; 2 Current address: University of California, Berkeley, CA, USA; 3 University of Copenhagen, Copenhagen, Denmark; 4 Department of Plant Biology, Carnegie Institution for Science, Stanford, CA, USA; 5 Current address: University of Massachusetts, Amherst, MA, USA; 6 Current address: Energy Biosciences Institute, Berkeley, CA, USA

**Keywords:** *Brachypodium distachyon*, Cell wall, NIR, Seed, Biofuel

## Abstract

**Background:**

The model grass *Brachypodium distachyon* is increasingly used to study various aspects of grass biology. A large and genotypically diverse collection of *B. distachyon* germplasm has been assembled by the research community. The natural variation in this collection can serve as a powerful experimental tool for many areas of inquiry, including investigating biomass traits.

**Results:**

We surveyed the phenotypic diversity in a large collection of inbred lines and then selected a core collection of lines for more detailed analysis with an emphasis on traits relevant to the use of grasses as biofuel and grain crops. Phenotypic characters examined included plant height, growth habit, stem density, flowering time, and seed weight. We also surveyed differences in cell wall composition using near infrared spectroscopy (NIR) and comprehensive microarray polymer profiling (CoMPP). In all cases, we observed extensive natural variation including a two-fold variation in stem density, four-fold variation in ferulic acid bound to hemicellulose, and 1.7-fold variation in seed mass.

**Conclusion:**

These characterizations can provide the criteria for selecting diverse lines for future investigations of the genetic basis of the observed phenotypic variation.

## Background

The investigation of natural variation is arguably one of the oldest fields in modern biology, and innumerable discoveries have been made by studying a wide variety of organisms. The advent of next generation technologies for whole-genome sequencing and the development of powerful genotyping techniques (e.g. genotyping by sequencing) enable researchers to saturate the genome of any organism with genetic markers. These new tools are especially powerful for associating natural phenotypic variation to specific DNA sequences and are leading to increased interest in natural diversity.

A relative of wheat, oat, and barley, *Brachypodium distachyon* was suggested as a model for the grasses in 2001 [1]. During the ensuing years, rapid progress has been made in developing research tools for this small annual grass, including efficient transformation methods [2-4]; a high-quality whole genome sequence [5]; large germplasm collections [4,6-8]; T-DNA mutant resources [9,10]; and more. (For a recent review see [11]). In addition, 54 diverse accessions have been resequenced (unpublished). Building on this foundation, the goal of this study is to gain an overview of natural diversity in the available *B. distachyon* germplasm and then to identify a core collection of lines for the further investigation of bioenergy and grain traits.

As a genetically tractable model, *Brachypodium* can be used to increase our understanding of the genes that control grass growth and cell wall composition. Biomass yield is a function of numerous factors including plant height, which is often positively correlated with biomass accumulation [12,13], and growth habit, which impacts the amount of space required between plants in the field. The density of the plant material is also a consideration, because denser biomass can be more efficiently transported to biorefineries [14] and might lead to higher biomass yield. Cell walls comprise the bulk of the plant biomass, and their composition determines the efficiency with which biomass can be converted into biofuel [15].

Although much of our knowledge about cell walls is derived from studies of the eudicotyledonous plant *Arabidopsis thaliana*[16], the composition of grass cell walls is quite distinct from that of dicot cell walls [17,18]. Major differences in the carbohydrate polymers of the primary cell walls include the type of hemicellulose (arabinoxylans in grasses and xyloglucans in dicots), the level of pectin (low in grasses and high in dicots), and the presence of mixed-linkage glucans (present in grasses and absent in dicots). In addition, grass primary cell walls contain significant amounts of phenolic compounds, some of which cross-link carbohydrate polymers, while the majority of dicot primary cell walls contain few or no phenolic compounds. Likewise, grass secondary cell walls contain relatively high levels of the phenolic compounds ferulic acid and ρ-coumaric acid [14]. Illustrating these compositional differences, quantification of the non-cellulosic monosaccharides extracted from mature stems revealed that grasses, including *Brachypodium* and the bioenergy species *Miscanthus*, had higher amounts of arabinose, but lower amounts of galacturonic acid and rhamnose when compared to *Arabidopsis*[19]. In addition to their relevance for biofuel production and animal feed, grass cell walls also play a major role in human nutrition, because they can be a large component of grains and have health benefits as the fiber fraction of grains such as oat and wheat [20].

Despite the biological and commercial importance of plant cell walls, it is difficult to precisely determine their composition [21,22]. Much of this difficulty is due to the extremely complex composite polymer nature of the cell wall [15]. Large spatial and temporal variation in cell wall composition further complicates our ability to reproducibly characterize this trait. In this context, spectroscopic techniques have been useful for surveying differences in cell wall composition, because many of the linkages and chemical groups contained in the cell wall contribute to the net signal. Near infrared (NIR) spectroscopy can be particularly useful because it is fast and requires little or no sample preparation [23,24]. A significant limitation of NIR analysis is that without samples of known composition to serve as calibration standards, it is impossible to identify specific compositional differences between samples. However, NIR spectroscopy can readily be used to determine if unknown samples differ in composition without identifying the exact chemical differences. Thus, NIR has been employed to identify plant cell wall mutants and to predict digestibility of forage grasses [25-27]. Another method to assess cell wall composition is to measure the intensity with which antibodies that recognize specific epitopes within cell wall polymers bind to cell wall samples. By combining the specificity of monoclonal antibodies (mAbs) with the high-throughput capacity of microarrays, it is possible to rapidly analyze large numbers of cell wall samples. This approach, known as comprehensive microarray polymer profiling or CoMPP, has been successfully applied to many diverse plants including grasses [28-30]. The primary limitations of this technique are that it is semi-quantitative, and mAbs are not available to study all epitopes. Nevertheless, CoMPP is a powerful tool for the high-throughput comparative analysis of large numbers of cell wall samples

In this paper we characterize several phenotypes in a large collection of *B. distachyon* germplasm and then select a core collection of 17 diverse lines for more extensive characterization. We observed significant variation in plant height, growth habit, flowering time, cell wall composition, and seed size. Our results demonstrate that the phenotypic diversity in the current *B. distachyon* germplasm collection is sufficient to allow researchers to better understand the genetic basis of traits relevant to the development of superior crops.

## Methods

### Plant lines

The full collection of lines contained 166 lines from Turkey, four lines from Iraq and one line from Spain. Inbred lines Bd1-1, Bd2-3, Bd3-1, Bd18-1, Bd21, Bd21-3 and the Turkish lines were described previously [3,4,6,7]. Line Bd30-1 was developed by Dr. David Garvin (USDA-ARS, St. Paul, MN, USA), from material collected in Spain by Dr. Antonio Manzaneda (University of Jaén, Spain).

### Plant growth conditions

Plants grown outside in the winter of 2008-2009 (experiment 1, Table 1) were planted in Supersoil potting mix (Rod McLellan Co., Marysville, OH) and fertilized once at planting with a time-release fertilizer containing micronutrients (Osmocote Plus 15-9-12, Scotts Co., Marysville, OH). For each line, approximately 30 seeds were sown in a 20 cm diameter plastic pot. The pots were set on raised metal benches with no shading or protection from rain. Supplemental water was applied as necessary to maintain even soil moisture until the plants began to senesce naturally.

**Table 1:** Summary of experiments conducted for phenotypic characterizations

**Experiment number**	**Description**	**Conditions**	**Parameters measured (No. of lines examined)**
**1**	Preliminary phenotypic survey	Outside, winter of 2008-2009	Height (171)
			Seed detachment (171)
			Architecture (171)
			Stem density (46)^1^
			NIR (39)^1^
**2**	Synchronization of flowering time	Growth chamber, with varying vernalization periods	Flowering time (16)^2^
**3**	Repeat of outdoor growing conditions	Outside, winter of 2010-2011, three trials planted over 24 days	Flowering time (17)
**4**	Flowering-time matched plants	Growth chamber with staggered vernalization times	Height (17)
			Stem density (17)
			CoMPP (15)^3^
**5**	Flowering-time matched plants for seed measurements	Growth chamber with staggered vernalization times	Seed size (16)^2^

^1^Smaller numbers of lines were examined for the labor-intensive stem density and NIR measurements.

^2^Bd1-1 was not included in this experiment because of its known long vernalization requirement.

^3^Lines BdTR2g and BdTR5i were omitted from the CoMPP analysis, because insufficient material was available.

Plants in growth chambers were grown as previously described [9] (experiments 2, 4 and 5, Table 1). Briefly, the potting soil consisted of a 1:2:3:3 mix of sandy loam, sand, peat moss and #3 vermiculite plus a time-release fertilizer with micronutrients (Osmocote Plus 15-9-12, Scotts Co., Marysville, OH). Growth chambers had 20 hours of illumination (150 μEm^-2^s^-1^) from fluorescent lights. The temperature regime was 24°C in the day and 18°C at night.

Plants grown outside in the winter of 2010-2011 (experiment 3, Table 1) were grown in the same soil as growth-chamber-grown plants. Weather data for the 2010-2011 outdoor trial were obtained from the Oakland International Airport weather station located 22 km from the lab in a similar microclimate (http://www.wunderground.com/history/airport/KOAK/2008/12/10/MonthlyHistory.html).

Vernalization was conducted by incubating imbibed seeds at 4°C for the required amount of time. Initially, seeds were planted in damp soil, and the pots were placed in the cold (experiments 1,2,4; Table 1). After noticing low germination rates for some lines, particularly BdTR2g, BdTR5i, and BdTR11i, we began removing the lemmas from seeds and sterilizing the seeds. Sterilization was accomplished by washing the seeds in 15% bleach plus 0.1% Triton-X 100 (Sigma-Aldrich, St. Louis, MO, USA) for 4 minutes, followed by two rinses in water (experiments 3,5; Table 1). The sterilized seeds were placed on moist paper towels in the cold, before being transferred to soil. This treatment improved overall germination. For vernalization periods longer than 3 weeks, pots were placed under continuous weak fluorescent lighting because seedlings emerged after approximately 3 weeks.

### Morphological measurements of plants

For plants grown outside in the winter of 2008-2009, the length of the longest culm in each pot was measured from the soil to the top of the seed head, omitting lemma hairs. The height of plants grown in the growth chamber was measured by uprooting the plants and measuring the length of the longest culm from the soil to the top of the seed head, excluding lemma hairs. Average heights of growth-chamber-grown plants were based on measurements of 3 to 24 individuals per line, with an average sample size of 16. Three lines had poor germination and were represented by three (BdTR2g and BdTR5i) or five individuals (BdTR11i). All height measurements were determined by straightening the stem at the time of seed harvest.

Stem density was determined by dividing the mass by the volume of the plant’s longest, intact, undamaged internode – usually the uppermost internode in the primary tiller. Internodes were photographed, and the width was measured at six points along the length of the internode using ImageJ software [31]. The average width was used to calculate the volume of the cylindrical internode.

### Near infrared spectroscopy

We used NIR to help us select lines that varied in cell wall composition. The uppermost two internodes (not including seed heads, leaf sheaths or nodes) of stems from fully senesced plants were harvested, cut into ~5 mm long pieces and placed into 2 ml impact-resistant tubes (#1420-9600, USA Scientific, Ocala, FL) containing one 6.2 mm and two 3.2 mm chrome steel grinding beads. The larger bead was placed between the smaller beads to ensure thorough grinding. Very small stems (<5 cm) were not used. The tubes were only filled about three-quarters full to allow for free movement of the steel balls. The stem segments in open tubes were oven-dried overnight at 70°C. After drying, the tubes were immediately capped and placed in a ball mill (MM400, Retsch, Haan, Germany) and ground for 12 minutes at 30 cycles per second. The ground stem material was then transferred to a glass slide and another glass slide was placed on top such that the powder was spread in a thin layer between the slides. A Field Spec Pro spectrometer equipped with the plant probe attachment (ASD, Boulder, CO) was then used to obtain an average spectrum from 35 readings. The spectra were then converted to a .dx format. Principal component analysis (PCA) was conducted using Win-DAS software [32]. The spectra were baseline-corrected and only the region from 1000 to 2400 nm was used for PCA.

### Seed measurements

Four groups of 25 seeds each were weighed separately and the mass divided by 25 to determine the average seed mass. Seeds were photographed, and seed length and width were measured using ImageJ software [31]. Ten seeds were measured for each parameter.

### CoMPP analysis

We analyzed stem samples from the core collection grown in the growth chamber with staggered vernalization (experiment 4, Table 1). However, BdTR2g and BdTR5i were omitted from the CoMPP analysis because insufficient material was available. CoMPP analysis was conducted as previously described [29]. Briefly, alcohol-insoluble reside was prepared by grinding stem samples to a fine powder in liquid nitrogen prior to extraction with ethanol and acetone. Initial trials using three previously used polysaccharide extraction solvents (cyclohexylenedinitrilotetraacetate (CDTA), NaOH and cadoxen [29]) indicated that the cadoxen step did not result in appreciable release of additional cell wall material after the NaOH extraction (not shown). Therefore, only CDTA and NaOH extractions were used for subsequent experiments. These extractions were printed at three dilutions (2-, 5-, and 25-fold). The microarrays were then probed separately with panels of monoclonal antibodies (mAbs); the resulting spot signals were quantified as described in [29]. All samples were run with four biological replicates and three dilutions.

To avoid artifacts due to signal saturation and zero values, one dilution was selected for each antibody for each extraction method. The appropriate dilution was selected by examining the raw data and finding the dilution that gave a strong yet unsaturated signal for most of the samples. The raw numbers were multiplied by the appropriate dilution factor and averages calculated for each point. The values were then normalized by assigning the highest individual reading a value of 1,000 and setting all negative values to zero.

## Results

### Morphological characterization of the full collection

**Figure 1:**
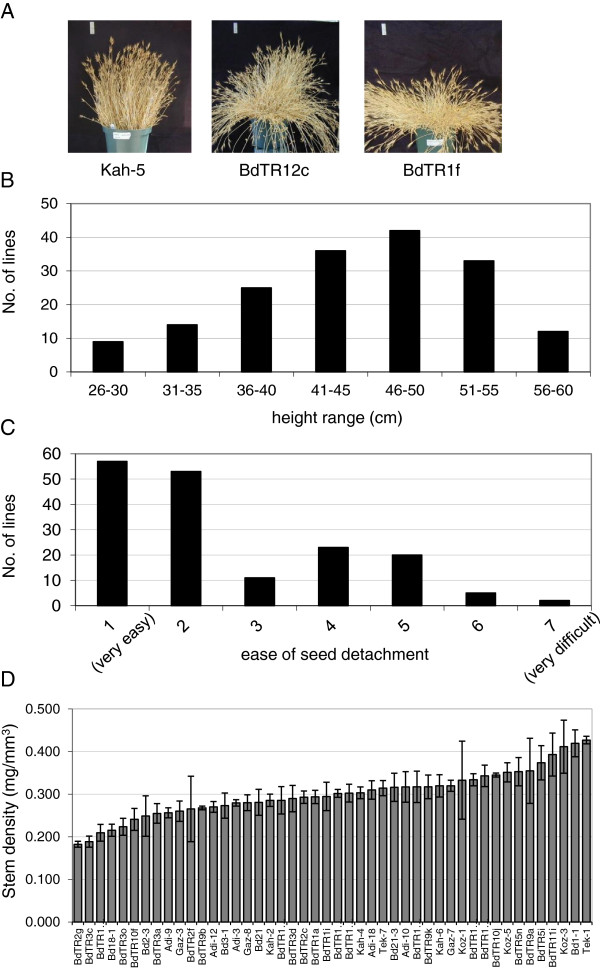
**Phenotypes of plants grown outside without controlled vernalization.** Multiple plants (approximately 30 per line) were grown outside in 20 cm pots widely spaced so the plants from adjacent pots would not touch one another as they grew. **(A)** Plant architecture varied considerably from erect like Kah-5 to intermediate or drooping like BdTR12c to almost prostrate like BdTR1f. The vertical white bar at the top left of each image represents 5 cm. **(B)** The height of the tallest plant in each pot was recorded, and heights were binned to graph the distribution. **(C)** The ease with which seeds could be removed from the stem was qualitatively determined and recorded on a scale from 1 (easy to detach) to 7 (difficult to detach). The distribution of the classes is presented. **(D)** Stem density was determined for 46 of the lines. Average stem density, measured for internodes from three to ten plants per line, is shown. Lines are arranged from the lowest to highest stem density. Error bars indicate the standard deviation. See Additional file 1: Table S1 for data on individual lines.

In order to obtain an overview of *Brachypodium* phenotypic diversity, morphological parameters including growth habit, stem length (height), ease of seed detachment, and stem density were evaluated. Growth habit in grasses is difficult to visualize from individual plants grown in a growth chamber. Thus, we only scored growth habit from groups of plants grown outside in large pots with ample surrounding space to allow them to take a natural form (experiment 1, Table 1). Growth habit varied from highly erect to nearly prostrate (Figure 1A, Additional file 1: Table S1, and Additional file 2: Figure S1). Stems of the most prostrate lines (*e.g.* BdTR1f) grew almost horizontally from the beginning and were not simply falling over under their own weight (Figure 1A). Some lines (*e.g.*BdTR12c) had long, flexible stems that grew up and then drooped over in a fountain-like effect, whereas others (*e.g.* Kah-5) remained upright throughout their lifecycle (Figure 1A). Bd21 and Bd21-3, commonly used as reference lines, were intermediate in their growth habit and were characterized as spreading and semi-erect, respectively. The maximum height in this experiment varied more than two-fold from 28 cm for Bd21 to 60 cm for Adi-6 and BdTR3e, with an average of 45 cm across all the lines (Figure 1B, Additional file 1: Table S1). Like Bd21, Bd21-3 – with a maximum height of 32 cm – was relatively short. The ease with which seeds fall off the stem (shattering) is a critical trait for cereals; mutations that prevent shattering have been critical in the domestication of all major grain crops [33]. The lines examined showed considerable variation in this essential trait, ranging from seeds that fell off when gently touched to two lines, Koz-3 and Tek-11, that retained seeds so tightly that they could not easily be detached by hand (Figure 1C, Additional file 1: Table S1). Based on these results and previously identified similarities in morphology, SSR genotype, and geographic origin [6,7], we could begin to narrow the collection of lines. We next measured stem density, a key biofuel trait, in a subset of 46 lines. Stem density differed more than two-fold from 0.18 mg/mm^3^ for BdTR2g to 0.43 mg/mm^3^ for Tek-1 (Figure 1D, Table 2). With stem densities of 0.28 and 0.32 mg/mm^3^ for Bd21 and Bd21-3, respectively, these reference lines were close to the overall average stem density of 0.30 mg/mm^3^.

**Table 2:** Stem density of plants grown outside in experiment 1

** Line**	**Stem density (mg/mm**^**3**^**)**^**1**^	**Standard deviation**
BdTR2g	0.182	0.007
BdTR3c	0.189	0.013
BdTR10c	0.209	0.020
Bd18-1	0.215	0.014
BdTR3o	0.224	0.020
BdTR10f	0.241	0.026
Bd2-3	0.249	0.047
BdTR3a	0.255	0.023
Adi-9	0.256	0.012
Gaz-3	0.260	0.024
BdTR2f	0.265	0.077
BdTR9b	0.268	0.004
Adi-12	0.270	0.013
Bd3-1	0.273	0.029
Adi-3	0.280	0.008
Gaz-8	0.280	0.018
Bd21	0.281	0.030
Kah-2	0.285	0.014
BdTR12c	0.286	0.032
BdTR3d	0.290	0.031
BdTR2c	0.293	0.015
BdTR1a	0.294	0.016
BdTR1i	0.295	0.033
BdTR13o	0.302	0.009
BdTR11d	0.302	0.021
Kah-4	0.303	0.014
Adi-18	0.310	0.022
Tek-7	0.314	0.018
Bd21-3	0.316	0.033
Adi-10	0.317	0.036
BdTR11h	0.317	0.037
BdTR9k	0.317	0.028
Kah-6	0.320	0.026
Gaz-7	0.320	0.013
Koz-1	0.333	0.092
BdTR13n	0.334	0.014
BdTR13c	0.343	0.025
BdTR10j	0.345	0.005
Koz-5	0.351	0.022
BdTR5n	0.353	0.033
BdTR9a	0.355	0.076
BdTR5i	0.374	0.040
BdTR11i	0.393	0.050
Koz-3	0.411	0.062
Bd1-1	0.419	0.032
Tek-1	0.427	0.009

^1^Stem density is based on measurements of internodes from 3-10 different plants per line. ANOVA (analysis of variance) testing obtained a *p*-value of 8.39 x10^-23^ for differences between groups.

### Near infrared spectroscopy

**Figure 2:**
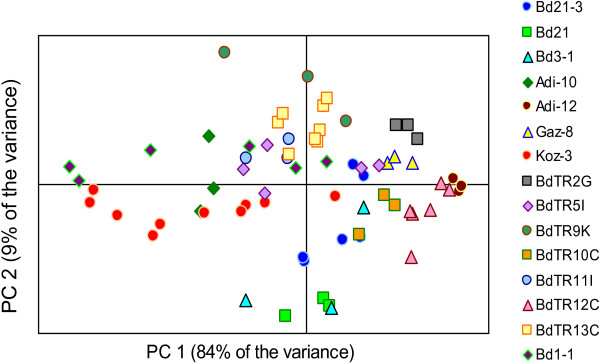
**Principal component analysis of NIR spectra from ground stems from the core collection of lines grown outside without controlled vernalization.** Samples from at least three plants per line were tested; each data point represents a single individual. Note the separation of groups along the first two principal components, PC 1 and PC 2. The percent variance explained by PC 1 and PC 2 is indicated.

To ensure that the core collection contained lines that differed in cell wall composition, we used NIR to analyze ground stems from 39 of the lines grown outside in the first experiment (Table 1). We performed principal component analysis (PCA) to visualize the differences in cell wall composition among the accessions and then used this information to inform our selection of the final core collection. The PCA results for 15 of the 17 lines in the core collection are presented in Figure 2. Two core collection lines, BdTR3c and Bd30-1, were not included because they were added to the core collection later and not analyzed by NIR. Although the lines tested by NIR exhibit differing amounts of within-group variability, samples from the same line generally cluster together. Overall, the PCA indicates that the NIR spectra of different lines diverge along the first two principal components, PC 1 and PC 2, which explain 84% and 9% of the variance in this data set (Figure 2). For example, while Bd21-3 samples are relatively centrally located along the PC 1 axis, the Koz-3 and Adi-12 lines separate along this axis. In another comparison, the lines BdTR12c and BdTR13c separate along both the PC 1 and PC 2 axes. Interestingly, PC2 distinguishes Bd21 spectra from those of Bd21-3. This result highlights the fact that, although Bd21 and Bd21-3 were derived from seeds collected at the same location in Iraq [3,4], these two lines are phenotypically, as well as genotypically [7], distinct.

### Selection of a core collection

For many applications it is impractical to use the full collection of 171 lines. Thus, it is desirable to select a small, highly diverse core collection. We used the phenotypic data described above, together with previously published genotypic and geographic data [7], to select a core collection of 17 lines. Twelve Turkish lines were chosen based on phenotypic information summarized in Additional file 3: Table S2. BdTR3c was included, even though we did not analyze it by NIR spectroscopy, because it had a maximum height of 59 cm – well above the average height of 45 cm for the 171 lines taken together. Bd30-1 is an inbred Spanish line which became available after the initial survey was finished; Bd30-1 was included to broaden the geographic distribution of the collection. Four well-characterized lines –Bd3-1, Bd21, and Bd21-3 from Iraq and Bd1-1 from Turkey – complete the core collection. Bd3-1 is commonly used for mapping, while Bd1-1 represents a distinctive clade of late-flowering lines [7]. Despite their similarity in many respects, both Bd21 and Bd21-3 were included, because Bd21 is the source of the reference genome and a parent of several recombinant inbred line (RIL) populations, and Bd21-3 is the parent of >20,000 T-DNA lines [9]; http://brachypodium.pw.usda.gov/TDNA/.

### Synchronization of flowering time in the core collection

*B. distachyon* lines differ considerably in flowering time when grown under the same conditions [6,34]. When grown outside without controlled vernalization, the earliest-flowering lines (Bd3-1, Bd21, and Bd21-3) flowered up to three months earlier than the latest flowering lines (*e.g.*Bd1-1 and Tek lines). These differences complicate the interpretation of experiments focused on fully mature plants for two reasons. First, late-flowering plants typically achieve a much larger biomass because many additional leaves, tillers and roots grow during the extended juvenile period. Second, since much of the lifecycle will have been completed at different times and plants in pots become pot-bound, plants with different flowering times may be subjected to significantly different environmental conditions over the course of development.

Fortunately, *B. distachyon* responds to vernalization at the seedling stage by accelerating flowering. Initial vernalization experiments conducted while creating inbred lines divided the lines into three broad groups [7]. The first group consists of four lines from Iraq (Bd21, Bd21-3, Bd2-3, and Bd3-1) that require a short vernalization of 3 weeks or less and require no vernalization at all under long-day conditions (>16 hrs. light) [4]. The second group of lines requires 3-5 weeks of vernalization and needs vernalization even under long days. The third group consists of lines that require very long vernalization (≥6 weeks) to initiate flowering. Most of these late-flowering lines form a genotypically distinct group based on SSR markers [7].

**Figure 3:**
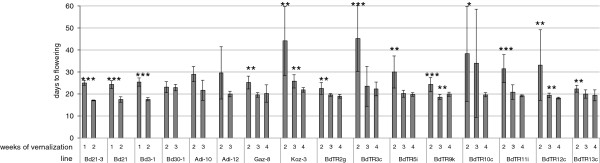
**Flowering time responsiveness to vernalization.** Seeds of inbred lines were cold-treated for the number of weeks indicated on the x-axis. Seeds were all sown on the same day and then grown under a 20-hour photoperiod. Flowering time was recorded for each plant when the first flower was visible to the naked eye. For calculation purposes, plants that had not yet flowered by the end of the experiment, 81 days after transfer to the growth chamber, were recorded as having a flowering time of 81 days. The average flowering time (y-axis) for each line and cold treatment is shown. Error bars indicate the standard deviation based on 13-20 plants. To test the statistical significance of observed differences, pairwise ANOVAs were performed for the longest cold treatment compared to each of the shorter cold treatments for each line. *: *p*-value < 0.05, **: *p*-value < 0.01, ***: *p*-value < 0.001.

To identify vernalization periods that promote synchronous flowering in the core collection, we cold-treated each line for different periods and measured the flowering time after shifting into a growth chamber with 20 hr days (experiment 2, Table 1, Figure 3). We combined stratification (treatment of imbibed seeds with cold) and vernalization by planting seeds in soil or imbibing seeds on paper towels and then placing them in a cold room with weak, continuous lighting. Under these conditions, seedlings emerge after approximately two to three weeks, but little vegetative growth occurs. Thus, differences in seedling size are negligible when the plants are moved to growth conditions. With one week of cold treatment, Bd21-3, Bd21, and Bd3-1 flowered within 24 to 25 days; in response to 2 weeks of cold treatment, they flowered within 17 to 18 days (Figure 3). For three other lines, Bd30-1, Gaz-8, and BdTR13c, a two-week-long cold treatment was sufficient to trigger flowering at approximately 24 days. ForAdi-10, BdTR2g, BdTR5i, BdTR9k, and BdTR12c, 3 weeks of cold treatment triggered flowering within 19 to 22 days, though in some cases – *e.g.* for BdTR2g – there was little difference between the 2- and 3-week-long cold treatments. For lines Adi-12, Koz-3, BdTR3c, BdTR10c, and BdTR11i, 4 weeks in the cold reliably triggered flowering approximately 3 weeks after transfer to long-day growth conditions. For this latter set of lines, inadequate vernalization not only prolonged the vegetative phase but also dramatically increased the variability of the flowering time transition. This variability is apparent in the large error bars for certain lines in Figure 3. For instance, Koz-3 plants cold-treated for 4 weeks had an average flowering time of 21.9 days, with a standard deviation of 1.1 days. In contrast, Koz-3 plants cold-treated for only 2 weeks flowered after 44.1 days ± 15.7 days. In fact, this range included two Koz-3 plants that flowered at 31 days, one plant that flowered at 73 days, and one plant that had not flowered by the end of the experiment at 81 days.

Plants subjected to excessive vernalization flowered almost immediately after moving from the cold room to the growth chamber and produced very short plants that were too small for most experiments. Thus, in addition to synchronizing flowering time, we selected a vernalization time that produced reasonably-sized plants (Table 3). This approach worked well for the lines in the two groups that required 5 weeks or less of vernalization. In practice, those lines all started to flower within a one-week period. This approach did not work as well with plants that required very long vernalization, because identifying a vernalization period that reliably resulted in reasonably-sized plants was difficult. For this reason, we excluded Bd1-1 from some experiments.

**Table 3:** Length of vernalization periods used to synchronize flowering of the core collection when grown under 20-hour days

**1 Week**	**2 Weeks**	**3 Weeks**	**4 Weeks**	**8 Weeks**
Bd21-3	Bd30-1	Adi-10	Adi-12	Bd1-1
Bd21	Gaz-8	BdTR2g	Koz-3	
Bd3-1	BdTR13c	BdTR5i	BdTR3c	
		BdTR9	BdTR10c	
		BdTR12c	BdTR11i	

**Figure 4:**
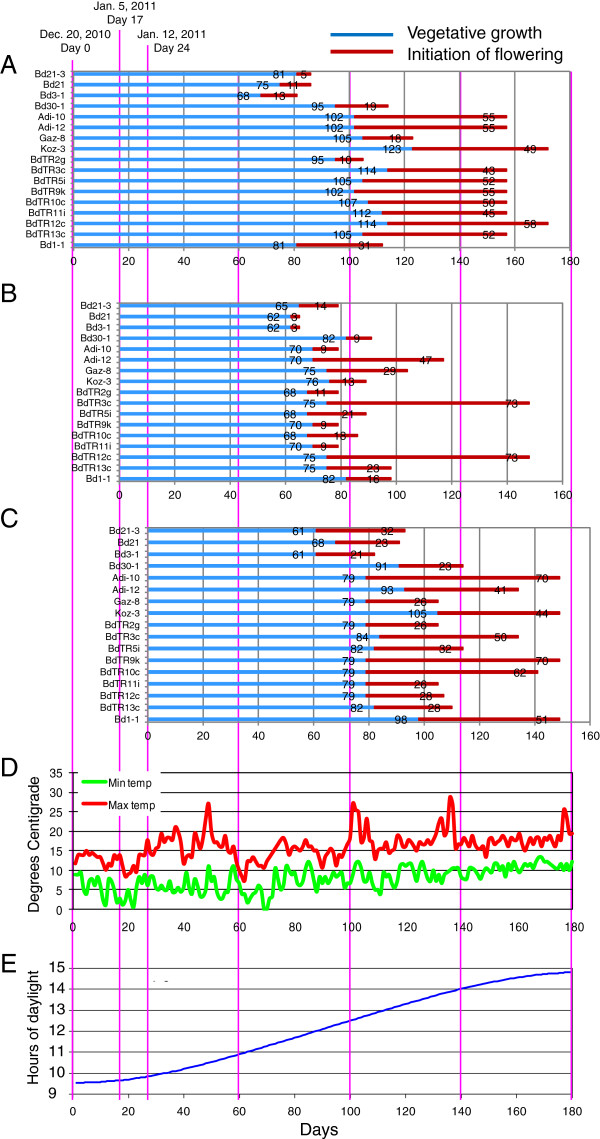
**Flowering time of plants grown outside.** Twenty seeds were planted in 20 cm pots and thinned to 15 plants per pot after germination. Three plantings were made on the dates indicated at the top of the figure. For all panels, the number of days is indicated on the x-axis. Vertical pink lines permit comparison across all x-axes. **(A-C)** The length in days of the vegetative phase is indicated by blue bars. The length of the flowering initiation phase, defined as the period from when the first plant in a pot flowered to the time when the last plant began flowering, is indicated by red bars. The length in days of each phase is indicated by the numbers on the bars. Names of lines are listed in the same order to the left of each graph. **(D)** Daily maximum and minimum temperatures, in degrees Celsius, recorded at a nearby weather station are indicated by red and green lines, respectively. **(E)** The blue curve indicates the daily daylength (y-axis) during the 180-day growing period (on the x-axis).

To further explore flowering time under more natural conditions, we grew the core collection in pots outside to repeat the conditions of experiment 1 (Table 1). In experiment 3 (Table 1), we conducted three trials initiated over a period of 24 days. The climate at our location (latitude: 38.048996, longitude: 122.140252) is similar to the Mediterranean climate where most of the lines were collected [6,7]. Typical winter temperatures are slightly above freezing with rare light frosts (Figure 4D). Figure 4A-C shows the lifecycle of the plants divided into vegetative and reproductive stages. Flowering time varied considerably among the lines, but most lines followed a similar trend in all three trials. The three early-flowering Bd lines, particularly Bd3-1, again flowered early in the outdoor trials. Those lines that started and/or finished flowering late outdoors (*e.g.* Adi-10, Adi-12, Koz-3, and BdTR12c) tended to be lines that had previously required three or four weeks of cold treatment to synchronize flowering in the growth chamber. Some of the within-line variation in the initiation of flowering may be due to insufficient vernalization, as observed for Figure 3. Interestingly, most of the lines in the first trial flowered after the second trial that was planted 17 days later. This result suggests that the initial 17 days of relatively cold temperatures and short days put the plants into a longer vegetative phase (Figure 4).

### Effect of environment on plant height and stem density in the core collection

**Figure 5:**
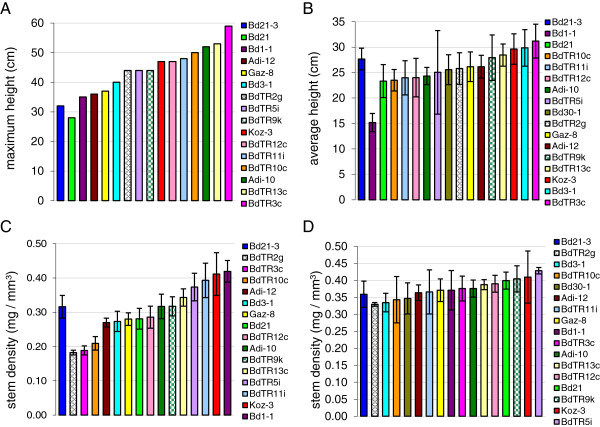
**Natural variation and environmental effects on plant height and stem density. (A)** Maximum height of plants grown outside. **(B)** Average heights of plants grown in a growth chamber with staggered vernalization to synchronize flowering. ANOVA *p*-value = 1 x 10^-17^ for differences between groups. **(C)** Average stem density of plants grown outside. ANOVA *p*-value = 1 × 10^-22^. **(D)** Average stem density of plants grown in a growth chamber with staggered vernalization to synchronize flowering. ANOVA *p*-value = 1 × 10^-4^. Lines are arranged with Bd21-3 first, as a reference, and then from the smallest to largest height **(A, B)** or stem density **(C, D)**. Bd30-1 became available after completion of the initial survey of lines grown outside (**A**, **C**, experiment 1) and was added for the later characterizations (**B**, **D**, experiment 4). Color-coding is indicated in the key and is the same for each line in all four panels. Error bars in **B**, **C**, and **D** indicate standard deviation.

Having observed large phenotypic differences in plants grown outside (Figures 1 and 2) and having determined that flowering time could be synchronized by varying the length of cold treatment (Figure 3 and Table 3), we were interested in investigating the extent to which the phenotypic variation persisted in flowering-time-matched lines. As mentioned above for plants grown outside, the stems of the earlier flowering lines were subjected to dramatically different environmental conditions than the stems of plants that flowered later. Thus, the large phenotypic differences observed may be due to a combination of genetic and environmental factors. We therefore grew the core collection in a growth chamber after staggered vernalization treatments, such that all lines flowered within approximately a one-week period (experiment 4, Table 1). The resulting plant heights and stem densities were compared to those of the plants initially grown outside (experiment 1, Table 1) without controlled vernalization (Figure 5). Overall, there was less variation in stem length and density between lines grown in the growth chamber; nevertheless, significant variation was still present, and several lines at the extremes tended to be at the extremes under both conditions (Figure 5).

Without controlled vernalization, plants were substantially taller: The core collection of lines attained heights of 28 to 59 cm outside versus average heights of 15 to 31 cm following staggered vernalization and cultivation in a growth chamber (Figure 5A, B). Thus, accelerating flowering through vernalization shortened the vegetative growth phase and resulted in less stem elongation prior to seed set. Bd1-1 provides a clear example of this general trend. Although a few centimeters taller than Bd21-3 when grown outside, Bd1-1 was 45% shorter than Bd21-3 when flowering was synchronized. This result suggests that the growth rate of the late-flowering line Bd1-1 is relatively slow and that, outdoors, it achieved a slightly greater height by undergoing stem elongation over a protracted period of time compared to the early flowering Bd21-3 line. For Bd21, Bd21-3, and Bd3-1, which have similar flowering times under various conditions (Figures 3 and 4), Bd21 was consistently the shortest of the three lines, Bd21-3 was intermediate in height, and Bd3-1 was the tallest. Both without and with controlled vernalization, BdTR3c was the tallest in the core set of lines, indicating that this height difference is at least partially under genetic regulation, rather than being simply the secondary effect of flowering time or environmental differences.

As observed for height, there was variation in the stem density of flowering-time-matched lines, although the magnitude of variation was smaller under the controlled conditions (Figure 5C, D). For plants with synchronized flowering times, the densest stems (0.43 mg/mm^3^) were only about 30% denser that the least dense stems (0.33 mg/mm^3^), compared to a difference of 230% for plants grown outdoors. Importantly, however, some lines showed similar trends under both conditions. For example, whether or not vernalization was staggered, BdTR2g was the least dense line, and Koz-3 was the second densest line.

### Comprehensive microarray polymer profiling in the core collection

To gain more molecular information about the differences between the stems of our core lines, we performed comprehensive microarray polymer profiling (CoMPP). The CoMPP technique provides information about the relative abundance of polysaccharide-borne epitopes across plant sample sets. Unlike glycosidic linkages and NIR spectra, epitopes can almost always be assigned with confidence to particular polysaccharide structures. However, it is important to note that the CoMPP spot signal values do not necessarily reflect the absolute amount of epitope present, because polysaccharide extractability may vary across the samples.

**Figure 6:**
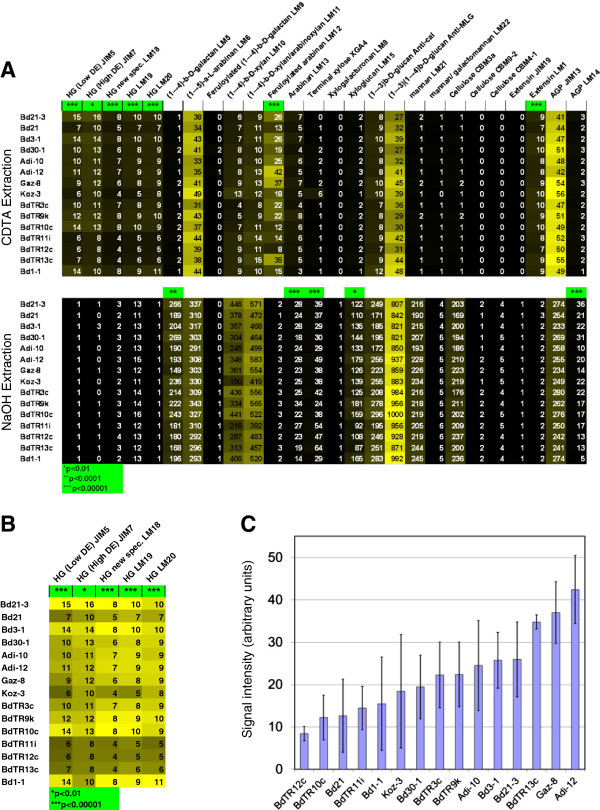
**CoMPP analysis of the core collection. (A)** Heatmap of the average antibody signal intensity from CDTA and NaOH extractions. For each extraction, the highest intensity (56 for CDTA and 1,000 for NaOH) signal was set to yellow and the lowest to black. The actual values are presented in each cell (with arbitrary units). Asterisks indicate significant differences between the lines, based on *p*-values determined by ANOVA for each antibody. One, two, and three asterisks indicate *p*-values below 0.01, 0.0001, and 0.00001, respectively. **(B)** The signal intensities from the CDTA extraction for all antibodies that recognize epitopes contained in pectin. Here yellow has been set to the highest signal intensity for each antibody individually. Note that similar trends are observed for all pectin epitopes for each line. **(C)** Signal intensity for the CDTA extraction probed with antibody LM12, which was raised against feruloylated arabinan, is plotted as a bar graph. The average signal intensity is shown for each line, with error bars indicating the standard deviation. ANOVA, *p* < 0.00001.

Heat maps of the average signal intensity for all antibodies are presented in Figure 6. ANOVA tests indicated that 12 of the 26 epitopes differed significantly between lines, 10 epitopes with *p*-values <0.0001 and two with *p*-values <0.01 (Figure 6). Interestingly, all five antibodies that recognize epitopes found in pectin followed the same pattern and identified significant differences between lines. To make the differences in pectin easier to visualize, a heat map with only the five pectin epitopes was created (Figure 6B). Another way to view the data is in bar graphs for the signal intensity from single antibodies. For example, the data for antibody LM12, which was raised against feruloylated arabinan, are graphed in Figure 6C. The four-fold variation is readily apparent in this format. Since this antibody recognized the ferulic acid moiety, the observed variation reflects overall feruloylation, not just feruloylated arabinans.

### Grain characteristics in the core collection

**Figure 7:**
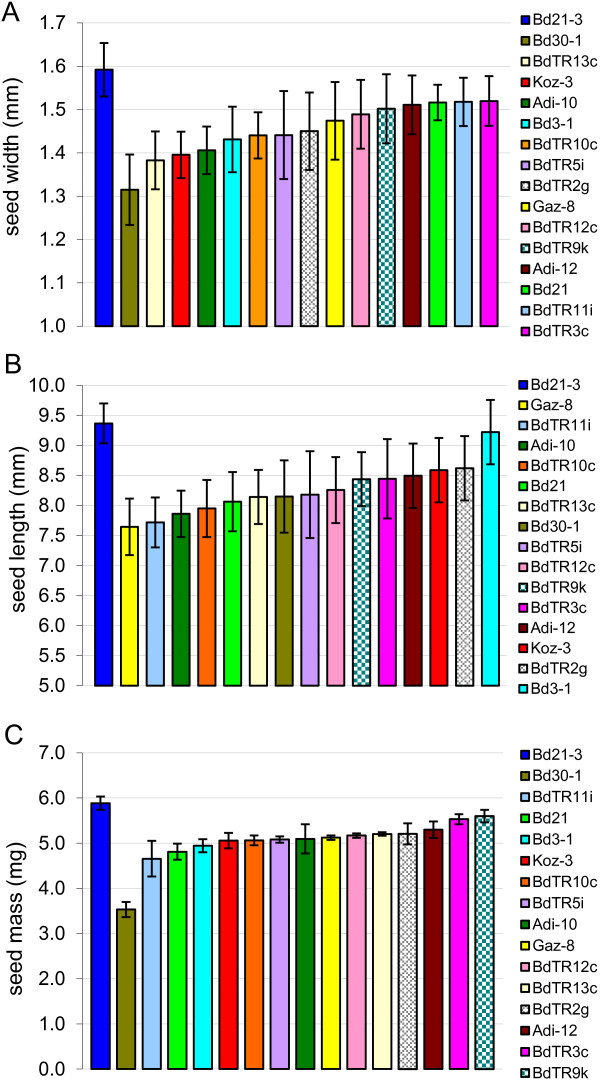
**Seed width, length, and mass measurements for natural accessions vernalized to synchronize flowering.** Averages and standard deviations are presented based on 10 seeds for **A** and **B** and four sets of 25 seeds for **C**. Note that for clarity, the y-axis begins at 1 mm in **(A)** and at 5 mm in **(B)**. To aid visualization, each line is represented by the same color in each graph and the order of the lines is reflected in the legends on the right. The color-coding is the same as in Figure 5. Our standard line Bd21-3 is always placed on the left and the other lines ordered from least to greatest average value. *P*-values for ANOVAs testing differences between lines were **(A)** 1 × 10^-15^, **(B)** 1 × 10^-12^, and **(C)** 1 × 10^-19^.

Because seeds are the most important part of the plant for grain crops, we also characterized the diversity of seed width, length, and mass in a flowering-time-matched set of the core collection (experiment 5, Table 1). Highly significant variation was observed in all three seed traits (Figure 7). Seed width raged from 1.3 to 1.6 mm, length ranged from 7.6 to 9.4 mm, and mass ranged from 3.5 to 5.9 mg/seed. Interestingly, the low mass of the lightest seeds, *e.g.* Bd30-1, seems to be due to their narrow width. The longest, widest, and heaviest seeds belonged to line Bd21-3, the line we use for transformation and mutagenesis [3].

## Discussion

As a step toward characterizing phenotypic variation in *B. distachyon*, we examined several traits in a diverse *B. distachyon* germplasm collection. We conducted an initial survey of growth habit, height, and seed shattering for 171 inbred lines (Figure 1). We also examined the density of stems and used NIR spectroscopy to infer cell wall differences in a smaller subset of the lines (Figures 2 and 3). Considerable variation existed for all traits examined. Using these results and previous genotypic data [7] we selected a core collection of 17 lines for more detailed characterization. Significantly, we have resequenced the genomes of all the lines in the core collection, and those sequences plus many more will be released shortly.

In order to conduct a robust phenotypic comparison of the lines in the core collection, we identified vernalization times that triggered nearly simultaneous flowering of the lines (Figure 3 and Table 3). This allowed us to remove flowering time as a variable and minimize the contribution of environmental differences toward phenotypic differences. Using the flowering-time-matched plants (experiment 4, Table 1), we again detected significant variation in all traits examined. Our assessment of height and stem density after senescence is relevant to the end-of-season harvesting employed for dedicated biofuel grasses and stover. By identifying lines that are both tall and dense, *e.g.* Koz-3, we are taking the first step toward identifying genotypes that lead to a favorable combination of these key traits.

Seed size is an important trait for grain crops. The largest average seed mass was 70% larger than the smallest (Figure 7). This variation in seed size is less than the 2.4-fold difference previously reported [7], because we did not include any lines from the group known to have small seed size (*e.g.* Bd1-1) due to their long vernalization requirement [7]. The fact that Bd21-3 and Bd30-1 represent the extremes for seed mass in the core collection, while having only slightly different vernalization requirements, makes these two lines good candidates for crossing in order to map the genetic basis of seed size.

Additionally, the use of NIR spectroscopy successfully allowed us to capture cell wall diversity when selecting the core collection of lines: In the CoMPP analysis, flowering-time-matched plants exhibited many differences in cell wall composition as measured by antibody binding (Figure 6). Differences detected by CoMPP included up to two-fold variation in signal intensity for all five antibodies that bound to various epitopes in pectin. While pectins are a small component of the grass cell wall [17,18], they are present in the middle lamella and play a key role in cell adhesion and division [35,36]. Thus, these observed compositional differences may result in developmental differences. Similarly, we observed a four-fold variation in the signal from an antibody that recognized ferulic acid bound to hemicellulose. Since ferulic acid contributes to the recalcitrance of biomass toward saccharification and fermentation to ethanol [37], this variation may be useful in tailoring the cell wall for use as a biomass feedstock.

The appearance of plants grown outside differs substantially from plants grown in greenhouses or growth chambers. This is not surprising because plants grown outside are subjected to higher light intensity, wind, and much greater environmental variability. In fact, it is difficult to observe differences in growth habit from plants grown in greenhouses and growth chambers. Thus, it is not surprising that the growth habit we observed in plants grown outside differs from a previous report on the growth habit of greenhouse-grown plants [6]. While scoring some phenotypes outside may be more agriculturally relevant, the environmental variability can also complicate efforts to identify genes controlling particular traits. Thus, lines with phenotypes that remain constant under varied environmental conditions are of particular interest. We measured height and stem density under two distinct conditions: outside without controlled vernalization and in growth chambers with staggered vernalization to synchronize flowering time. While the phenotypic differences were less dramatic in growth-chamber-grown plants, several lines followed the same trends under both conditions. For example, BdTR3c was consistently tall; BdTR2g had the least dense stems, and Koz-3 had the second densest stems under both conditions. Overall, cataloging phenotypes and especially identifying lines with contrasting phenotypes provide a foundation for further studies investigating the genetic factors regulating these phenotypes. Whether the trait is growth habit, flowering time, seed size, or abundance of cell wall epitopes, phenotypic data of the type presented here can, for example, inform the choice of lines for the generation of RIL populations, genome resequencing, and genome-wide association studies.

## Conclusion

In summary, we observed a significant amount of natural variation in the wild grass *B. distachyon* in traits relevant to grain and biomass crops. Since *B. distachyon* is amenable to experimental manipulation and genetic analysis, relatively rapid identification of the genes controlling this variation is feasible. These genes can then be used to improve crops through biotechnology, as well as by guiding the mining and deployment of natural variation in the crops themselves. In this context, it is important to note that *B. distachyon* has not experienced a population bottleneck as have the species grown for grain in the course of their domestication. Thus, in addition to identifying genes more rapidly, *B. distachyon* may contain genes and natural variation that are simply not present in domesticated cereals. Although a population bottleneck is not a problem with most of the grasses likely to be deployed as the first large-scale biomass crops (*e.g.* switchgrass), their large size, complex genetics and long generation times make identifying the responsible genes extremely difficult. Thus, for both grains and biomass crops, the identification of genes that control natural diversity in *B. distachyon* could prove extremely useful.

## Competing interests

The authors declare that they have no competing interests.

## Authors’ contributions

LT designed and conducted morphological and NIR experiments, drafted the manuscript; JUF, ADF and WGTW designed and conducted the CoMPP experiment; MAS and TKR developed the NIR method and interpreted data; JPV conceived the project, helped design experiments, interpreted data and help draft the manuscript. All authors read and approved the final manuscript.

## Supplementary Material

Additional file 1: Table S1 Preliminary survey of growth habit, height, and seed detachment phenotypes for 171 *B. distachyon* lines grown outside without controlled vernalization, experiment 1. Click here for file

Additional file 2: Figure S1 Images of lines grown outside without controlled vernalization, experiment 1. Click here for file

Additional file 3: Table S2 Summary of phenotypes for the core set of natural accessions grown outside without controlled vernalization, experiment 1. Click here for file
