# Supplementary material for: Selection and phenotypic characterization of a core collection of Brachypodium distachyon inbred lines
Source: BMC Plant Biol. 2014 Jan 14;14:25. doi: 10.1186/1471-2229-14-25 (PMC3925370; doi:10.1186/1471-2229-14-25)
Supplement: Additional file 2: Figure S1 — Images of lines grown outside without controlled vernalization, experiment 1. [file 1471-2229-14-25-S2.pptx]

## Slide 1
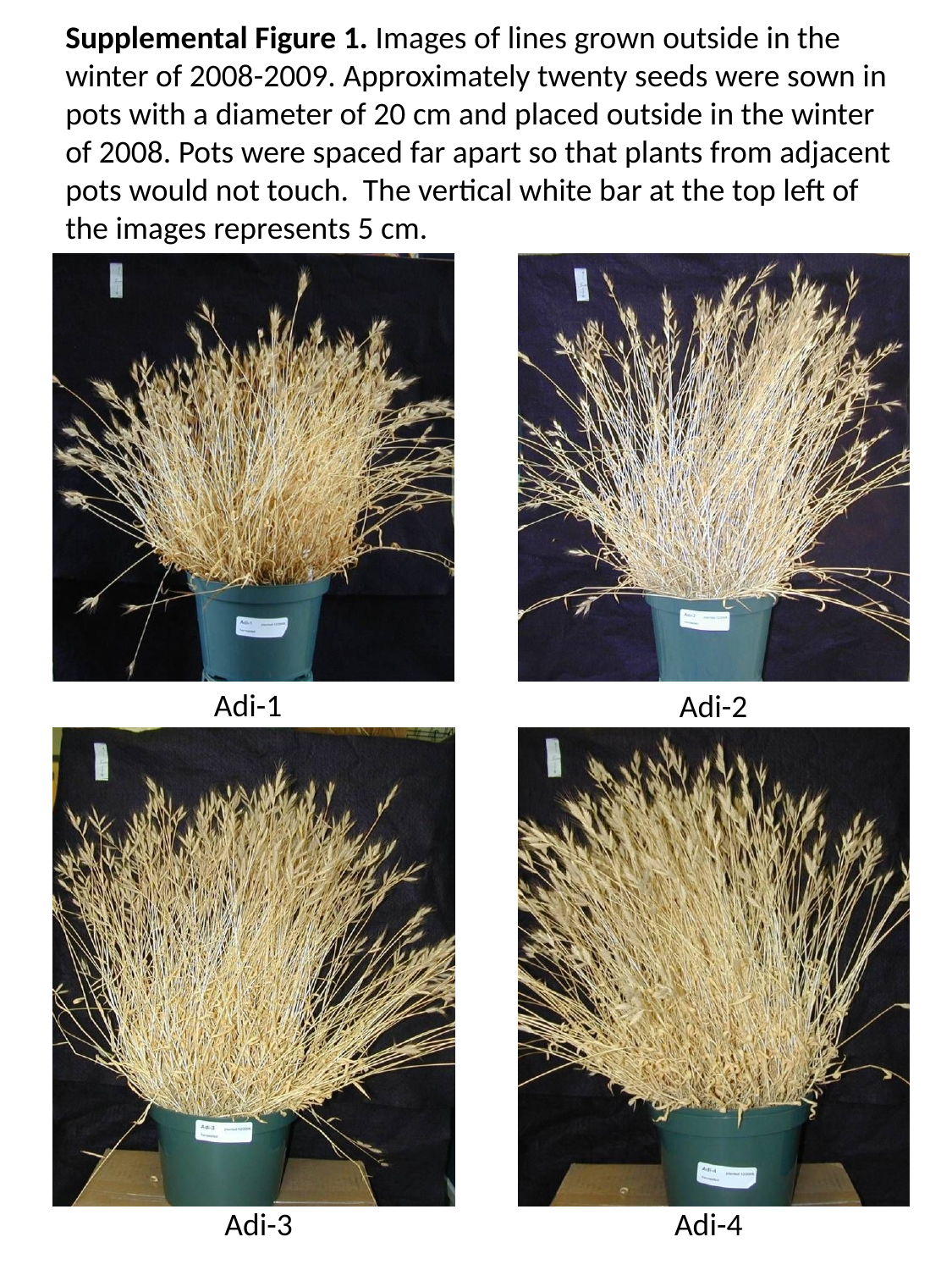

Supplemental Figure 1. Images of lines grown outside in the winter of 2008-2009. Approximately twenty seeds were sown in pots with a diameter of 20 cm and placed outside in the winter of 2008. Pots were spaced far apart so that plants from adjacent pots would not touch. The vertical white bar at the top left of the images represents 5 cm.
Adi-1
Adi-2
Adi-3
Adi-4

## Slide 2
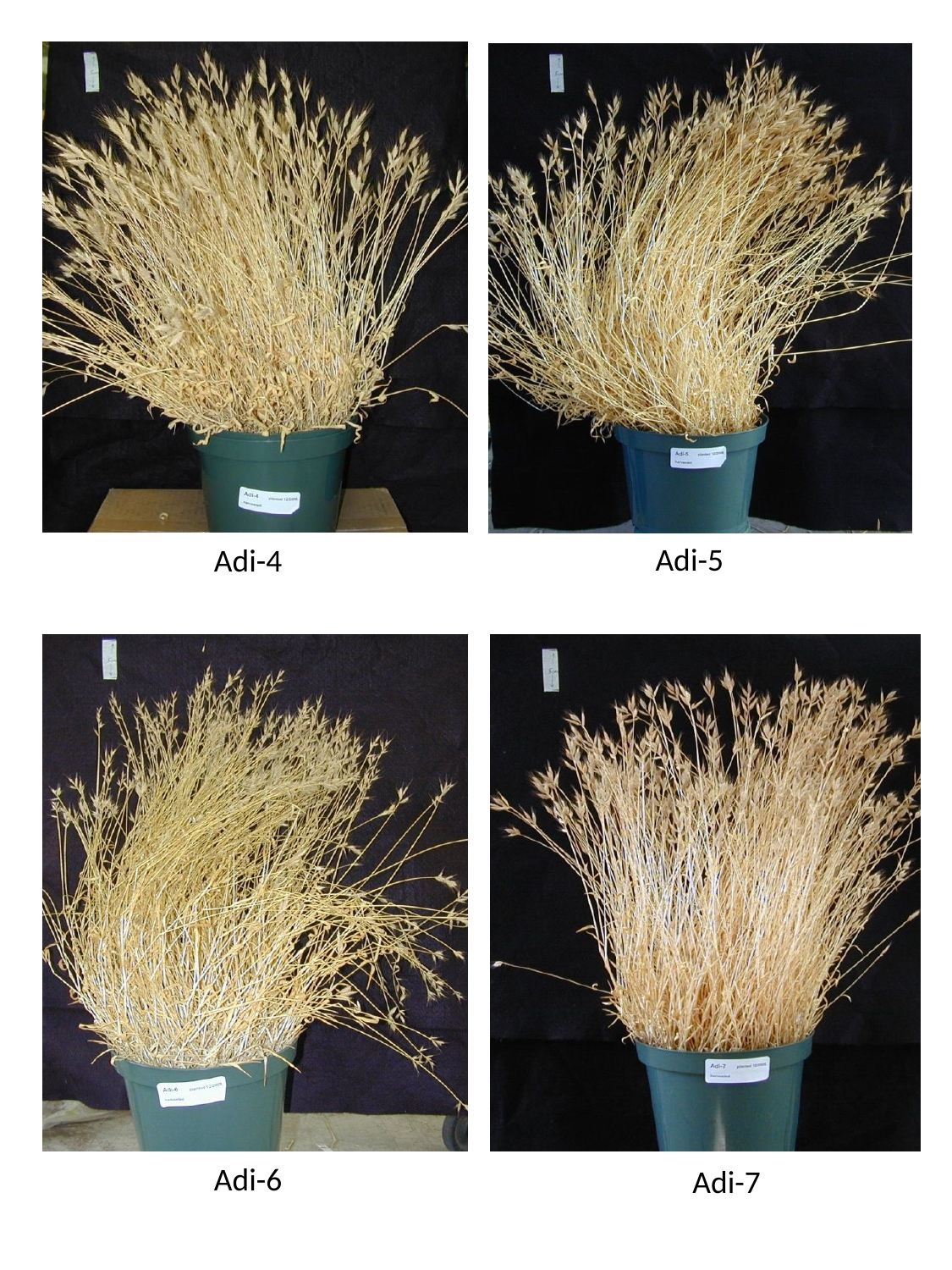

Adi-5
Adi-4
Adi-6
Adi-7

## Slide 3
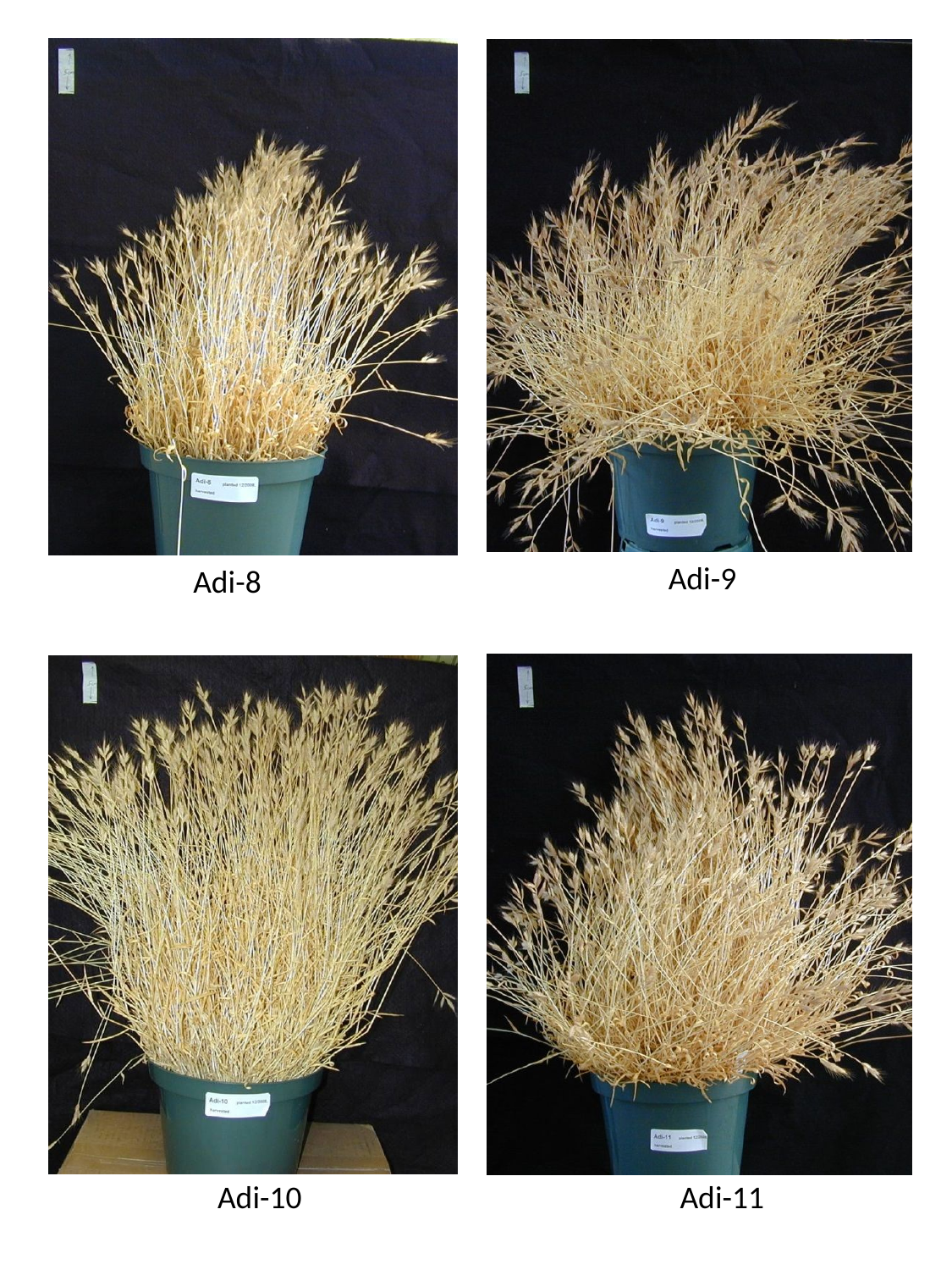

Adi-9
Adi-8
Adi-11
Adi-10

## Slide 4
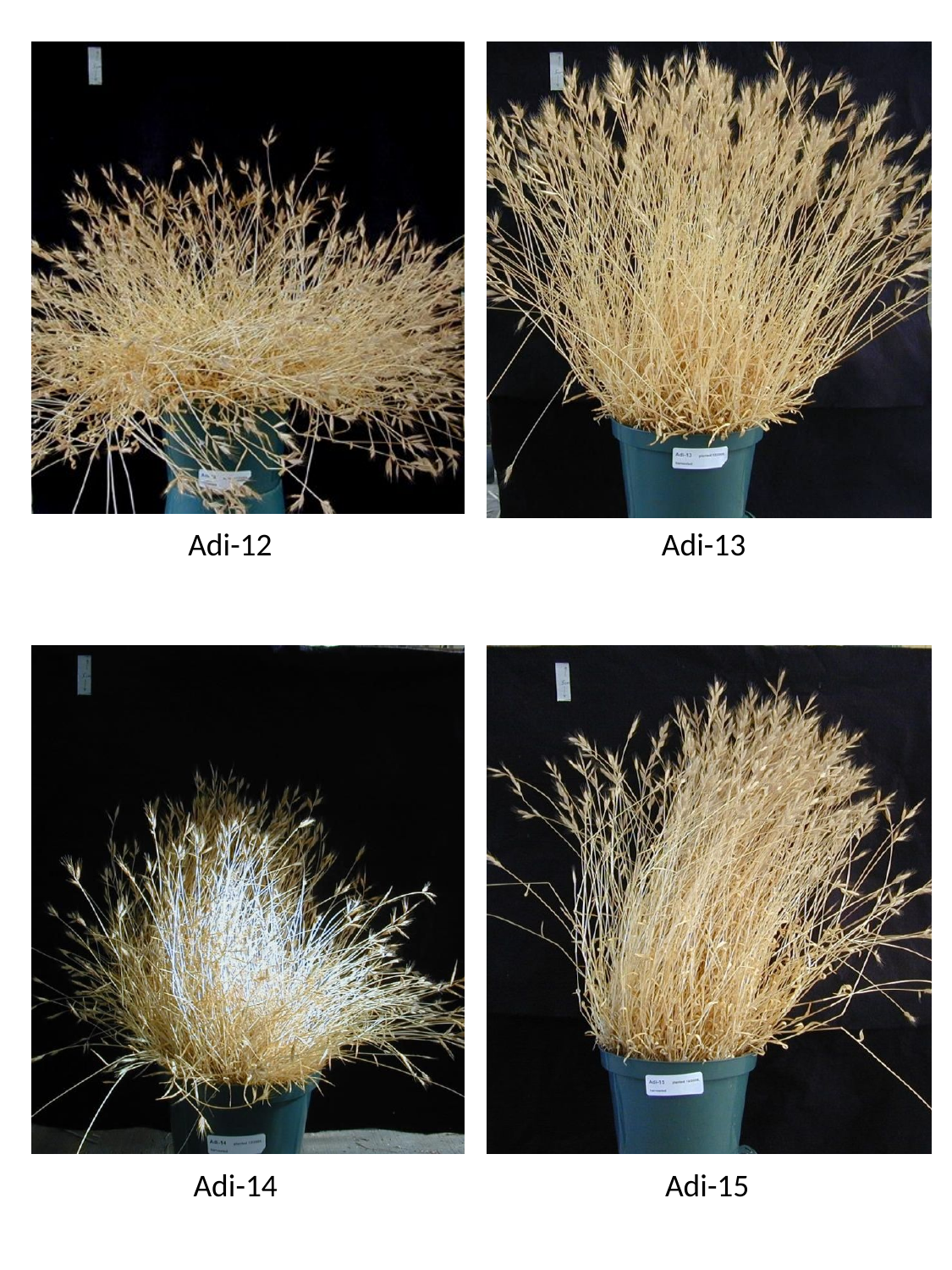

Adi-12
Adi-13
Adi-14
Adi-15

## Slide 5
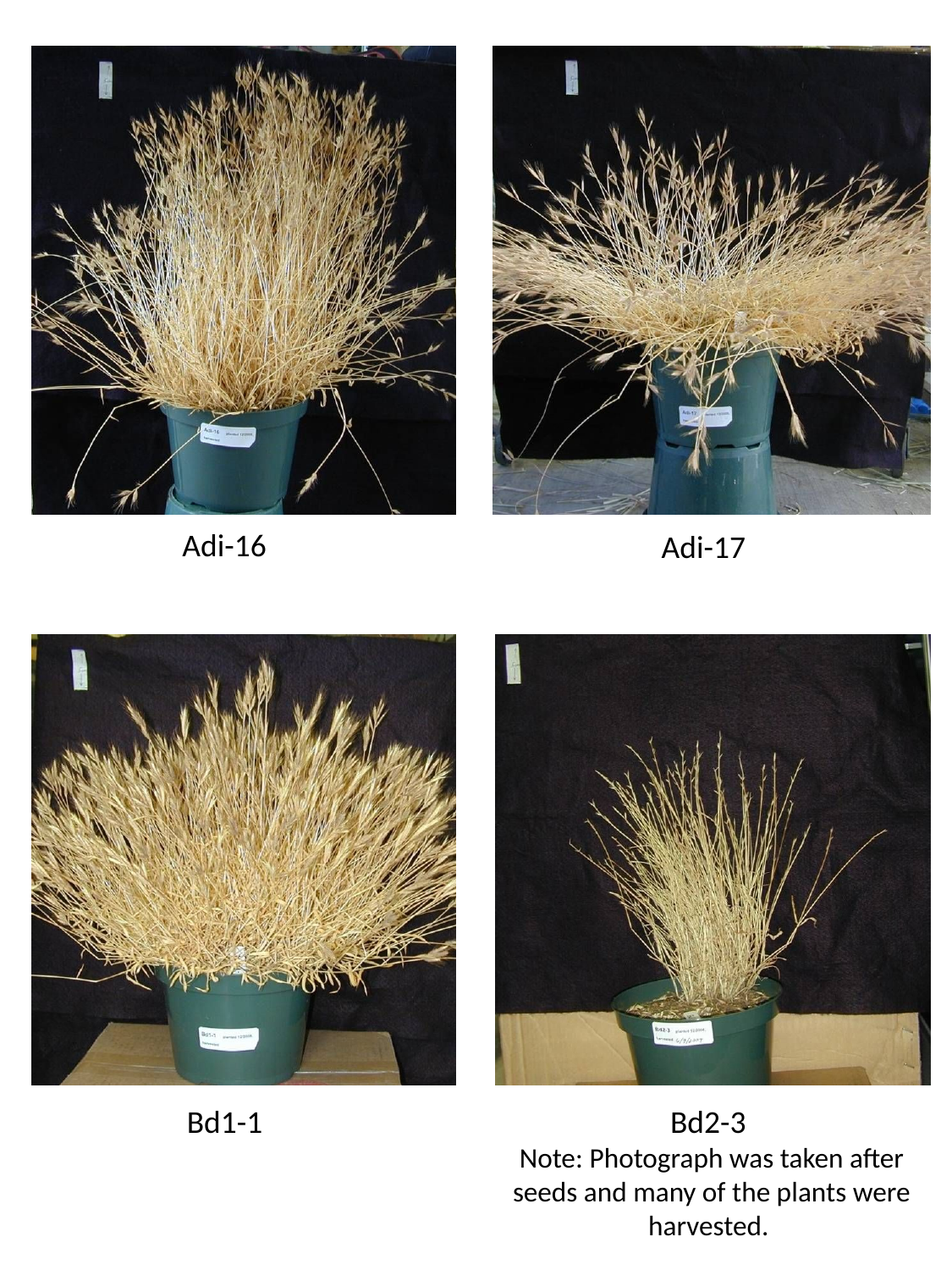

Adi-16
Adi-17
Bd1-1
Bd2-3
Note: Photograph was taken after seeds and many of the plants were harvested.

## Slide 6
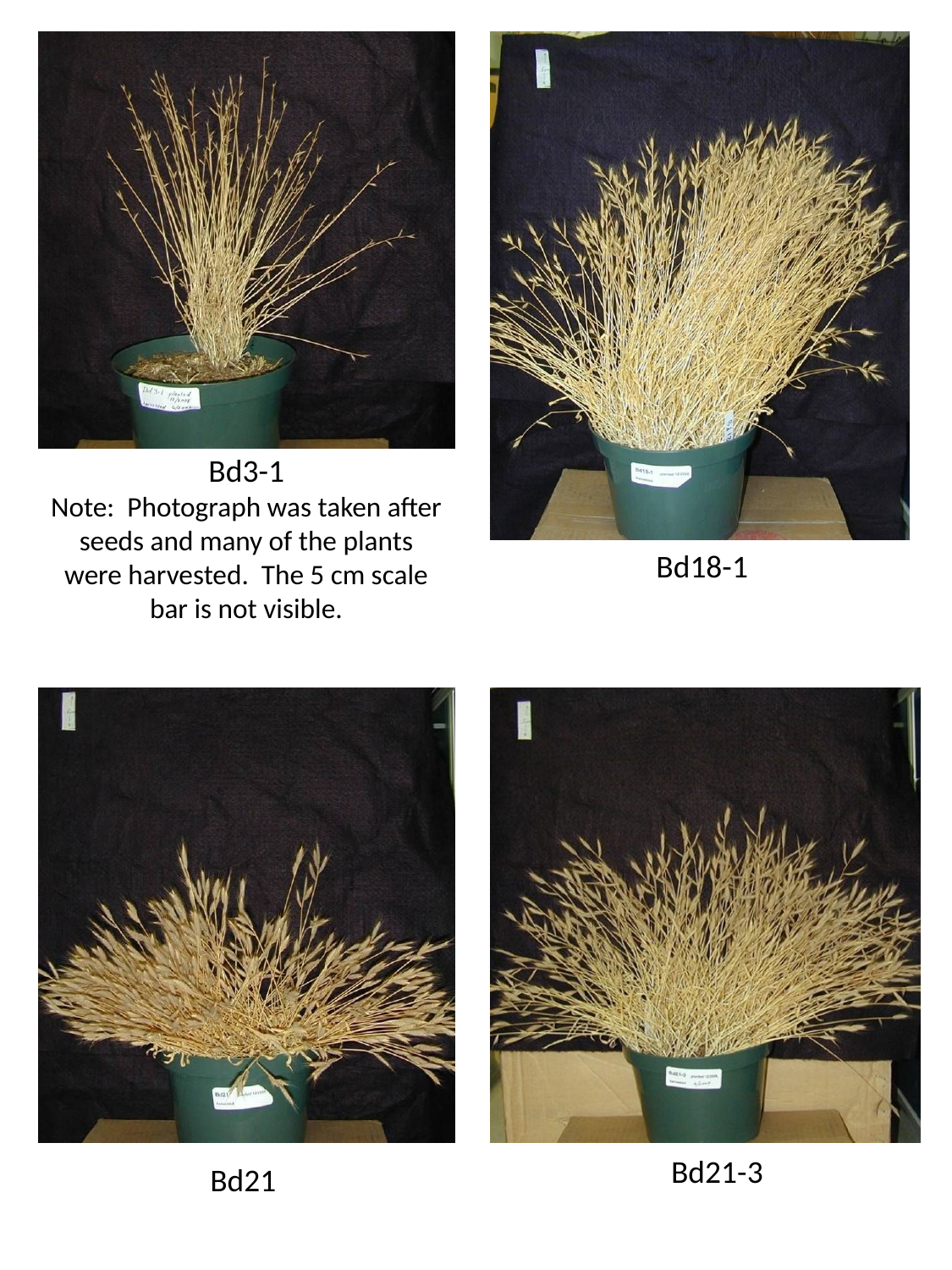

Bd3-1
Note: Photograph was taken after seeds and many of the plants were harvested. The 5 cm scale bar is not visible.
Bd18-1
Bd21-3
Bd21

## Slide 7
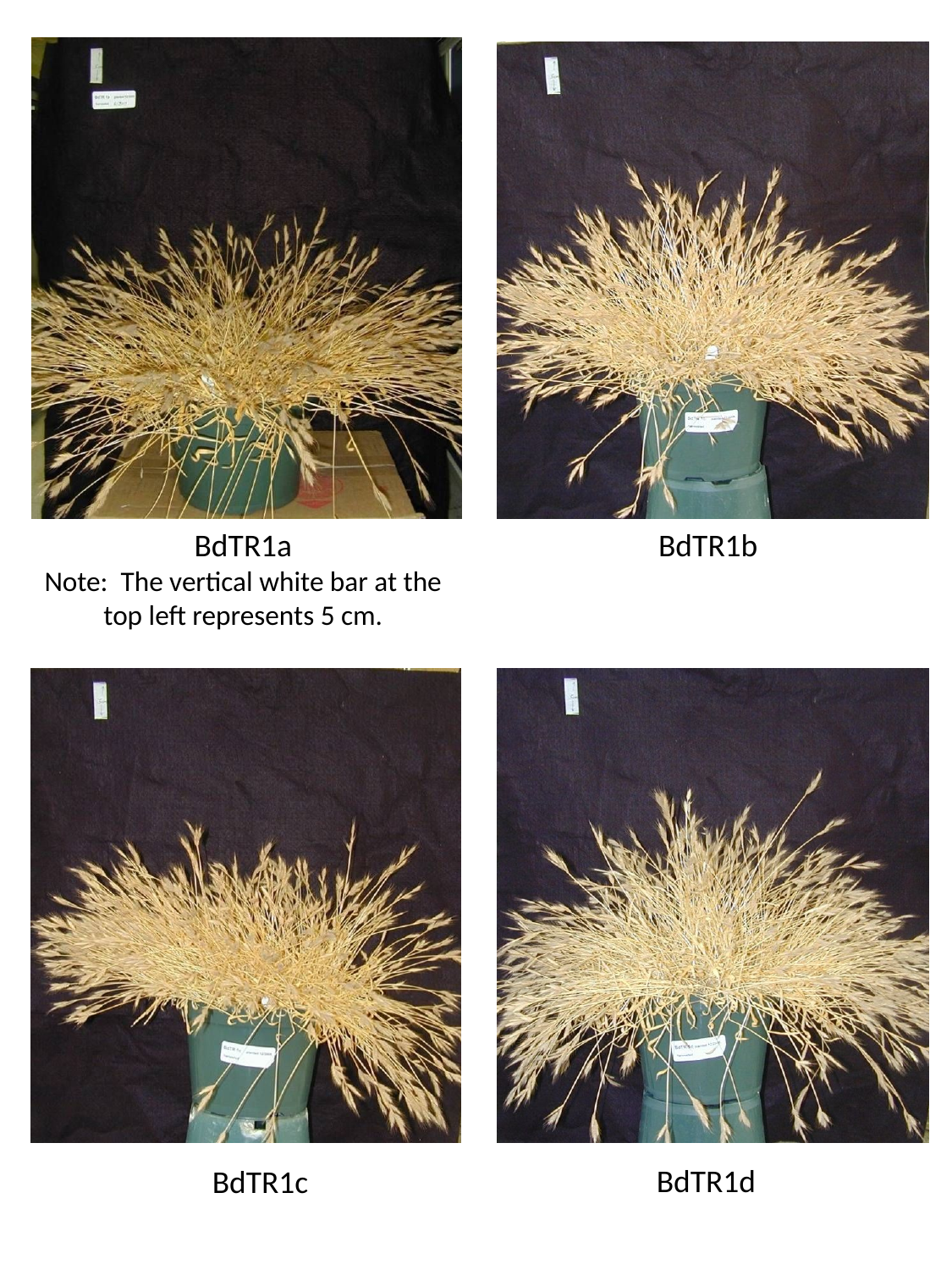

BdTR1a
Note: The vertical white bar at the top left represents 5 cm.
BdTR1b
BdTR1d
BdTR1c

## Slide 8
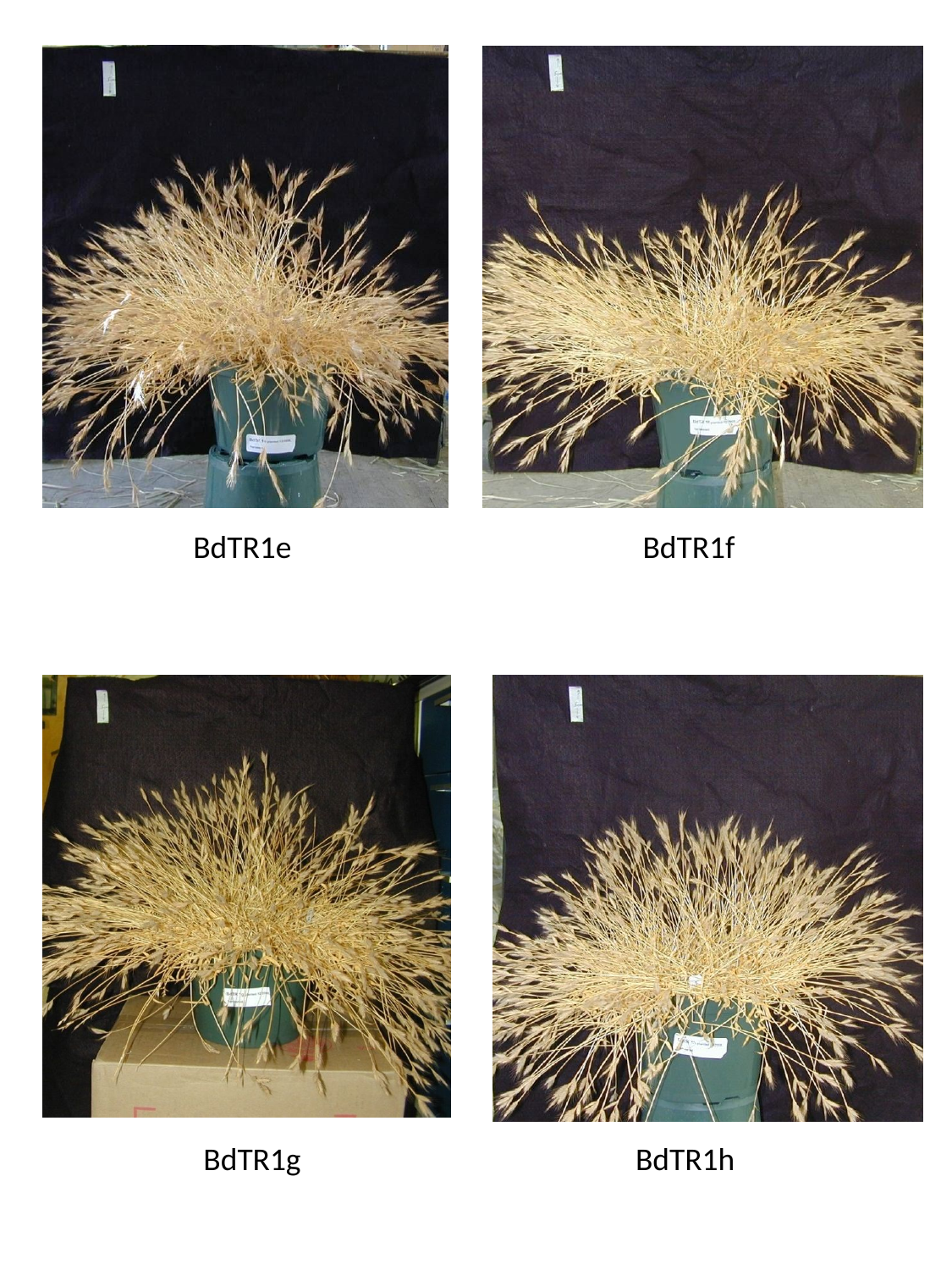

BdTR1e
BdTR1f
BdTR1h
BdTR1g

## Slide 9
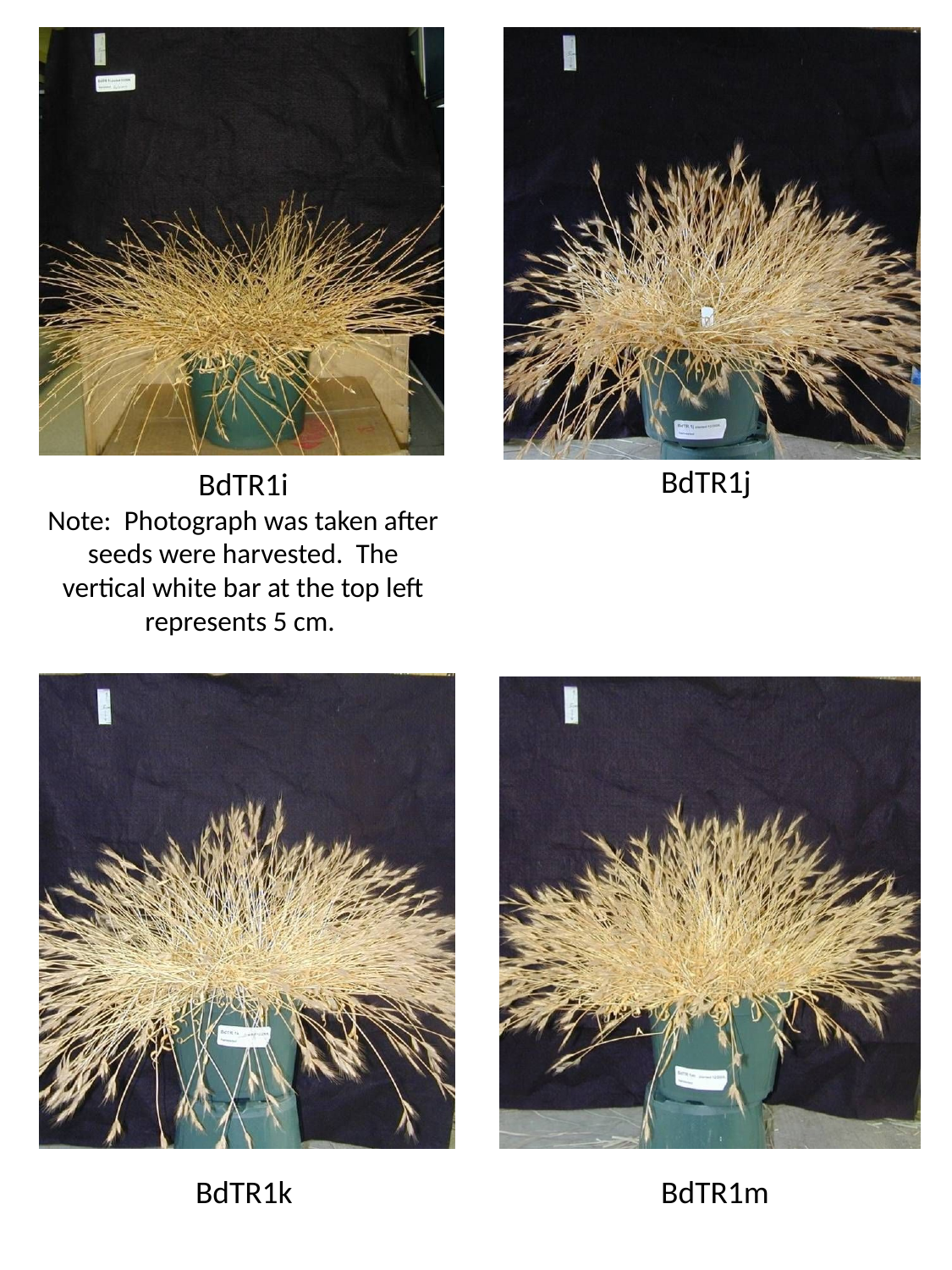

BdTR1j
BdTR1i
Note: Photograph was taken after seeds were harvested. The vertical white bar at the top left represents 5 cm.
BdTR1k
BdTR1m

## Slide 10
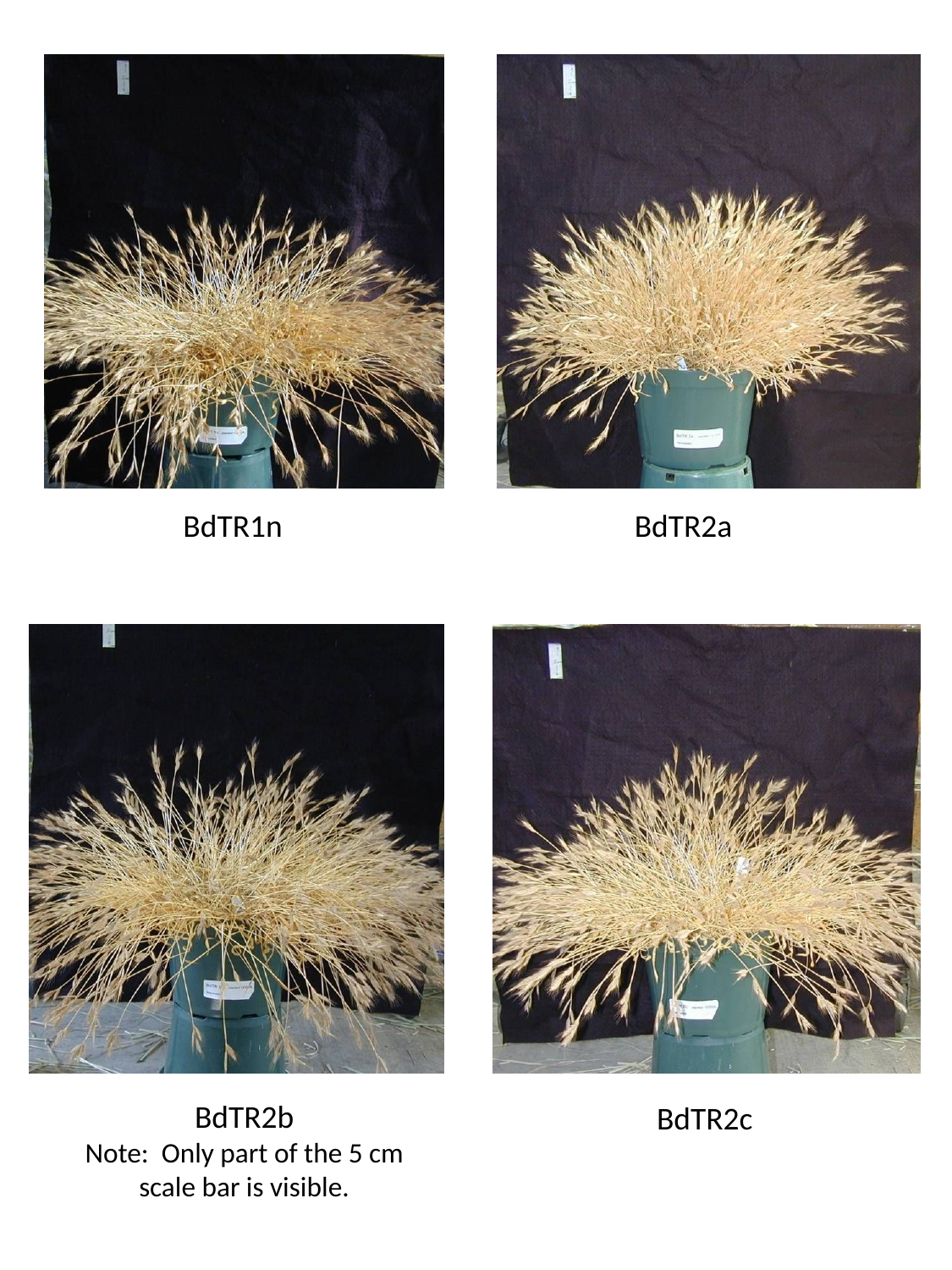

BdTR1n
BdTR2a
BdTR2b
Note: Only part of the 5 cm scale bar is visible.
BdTR2c

## Slide 11
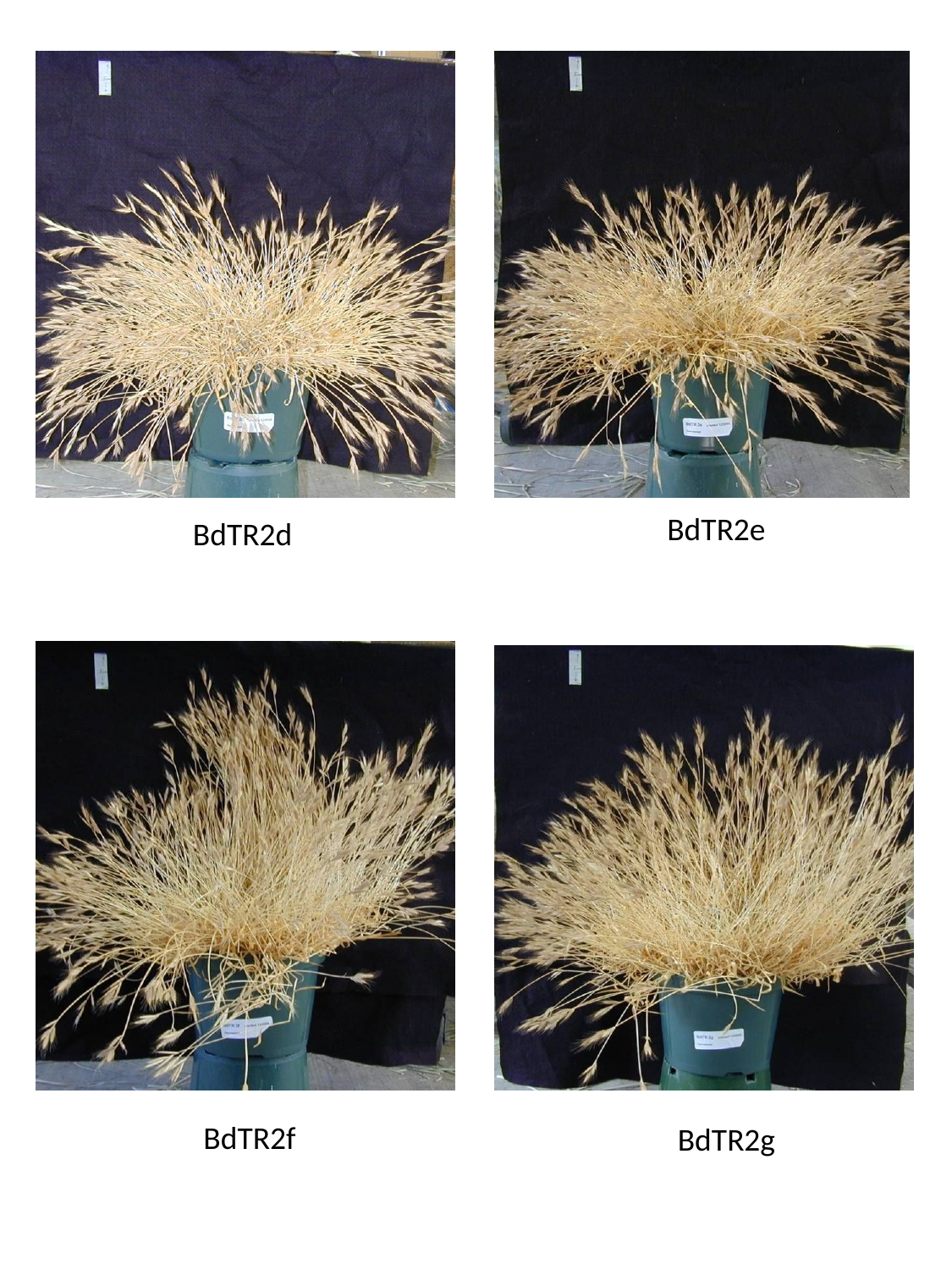

BdTR2e
BdTR2d
BdTR2f
BdTR2g

## Slide 12
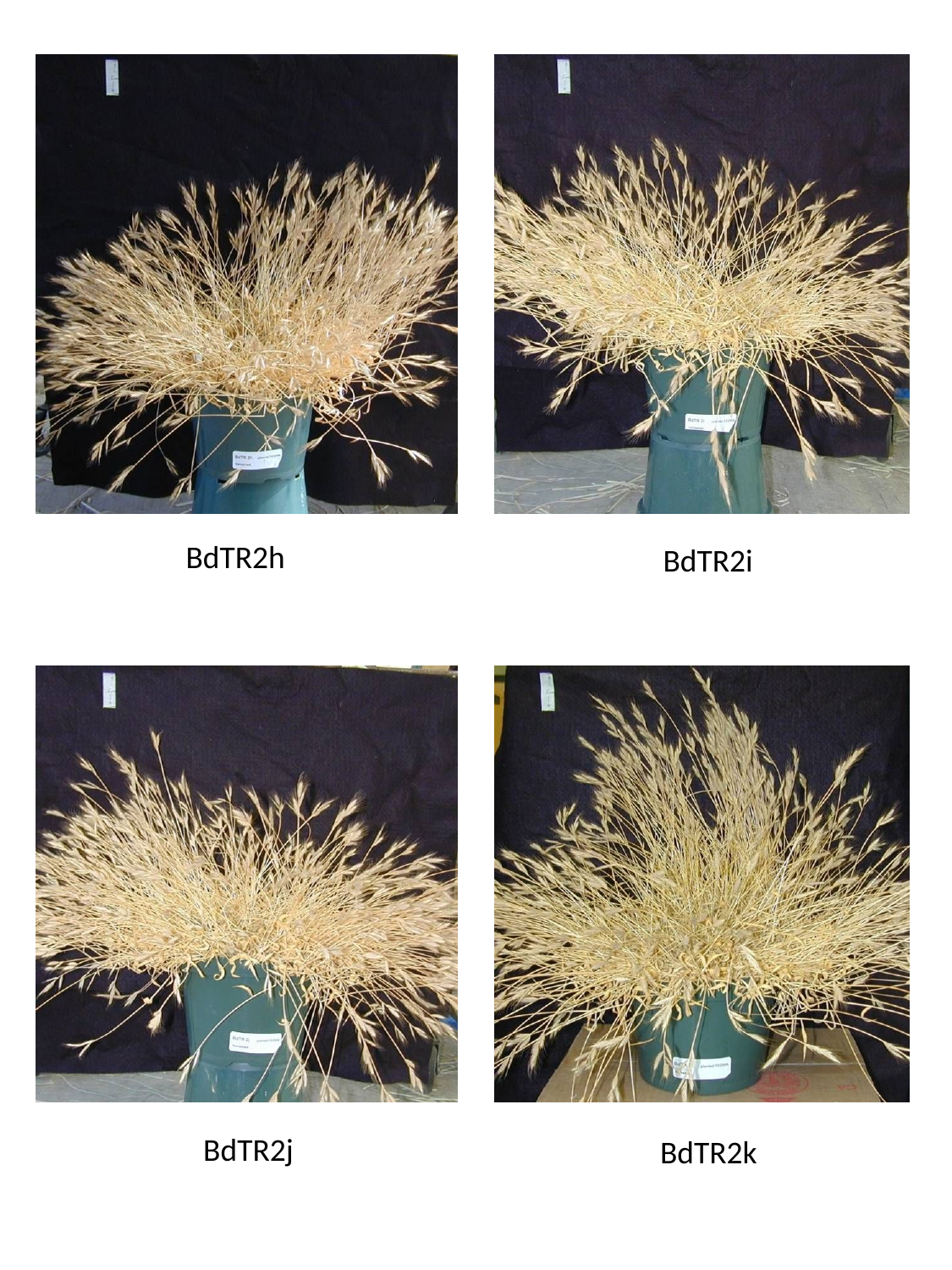

BdTR2h
BdTR2i
BdTR2j
BdTR2k

## Slide 13
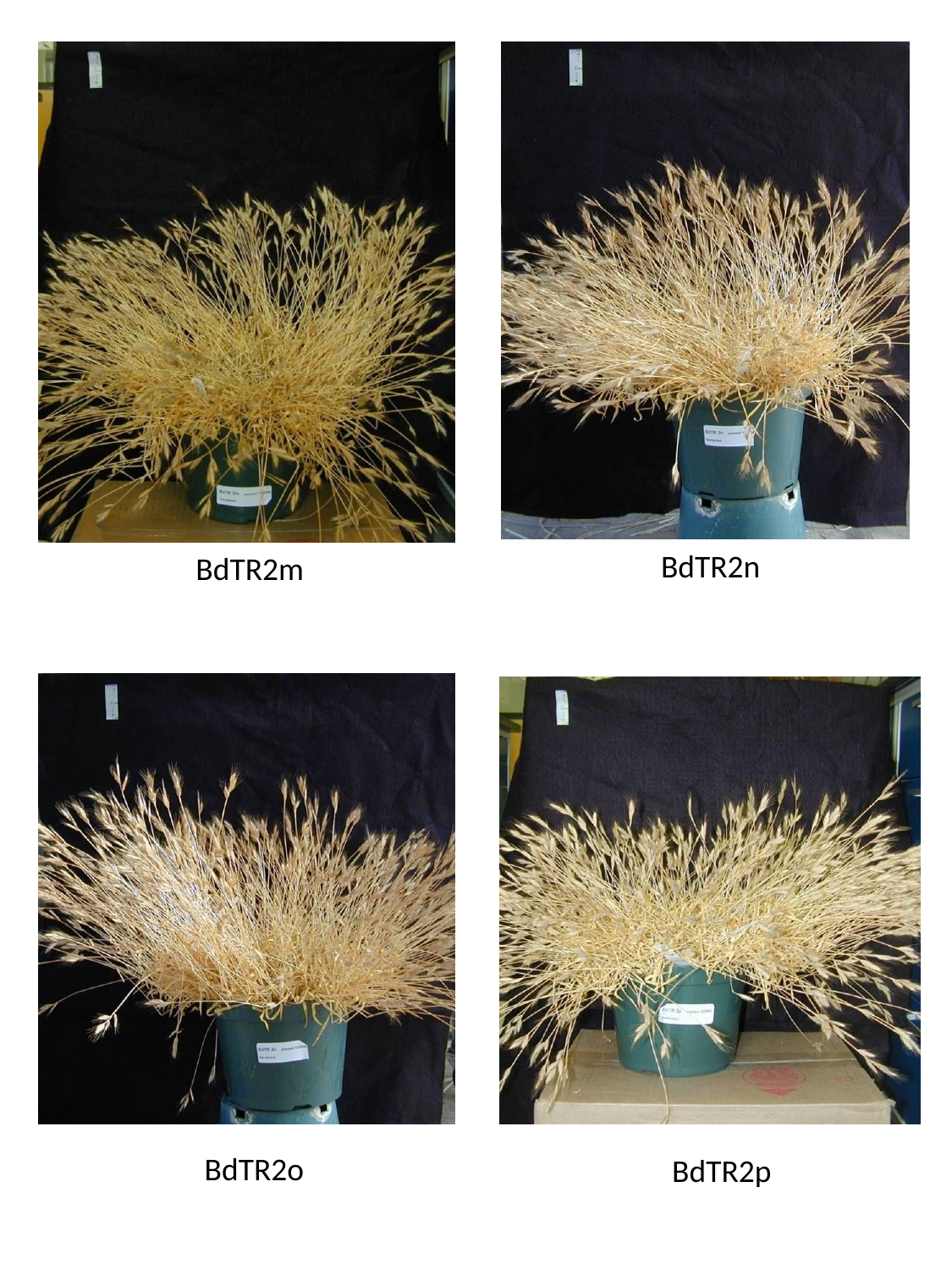

BdTR2n
BdTR2m
BdTR2o
BdTR2p

## Slide 14
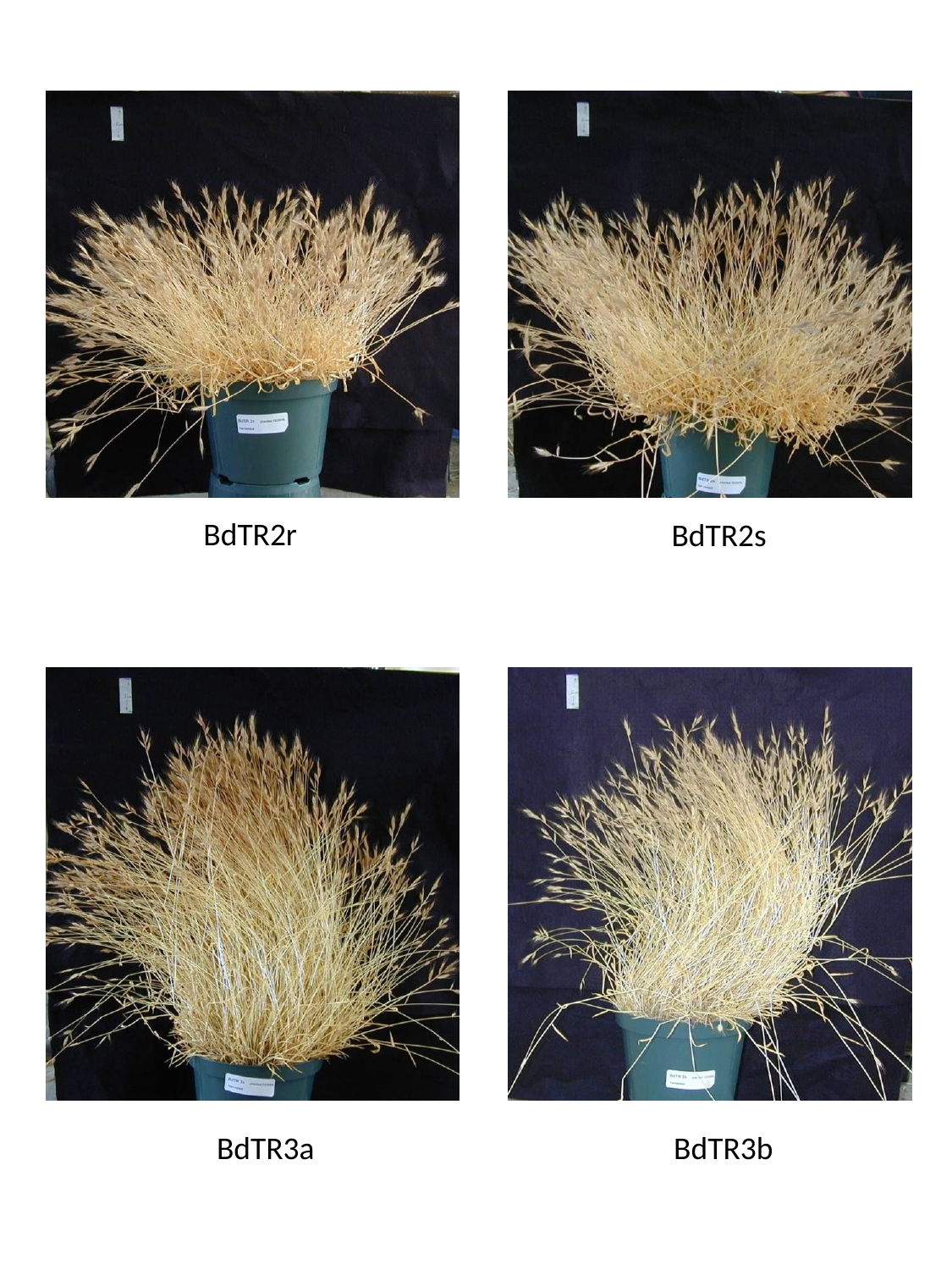

BdTR2r
BdTR2s
BdTR3a
BdTR3b

## Slide 15
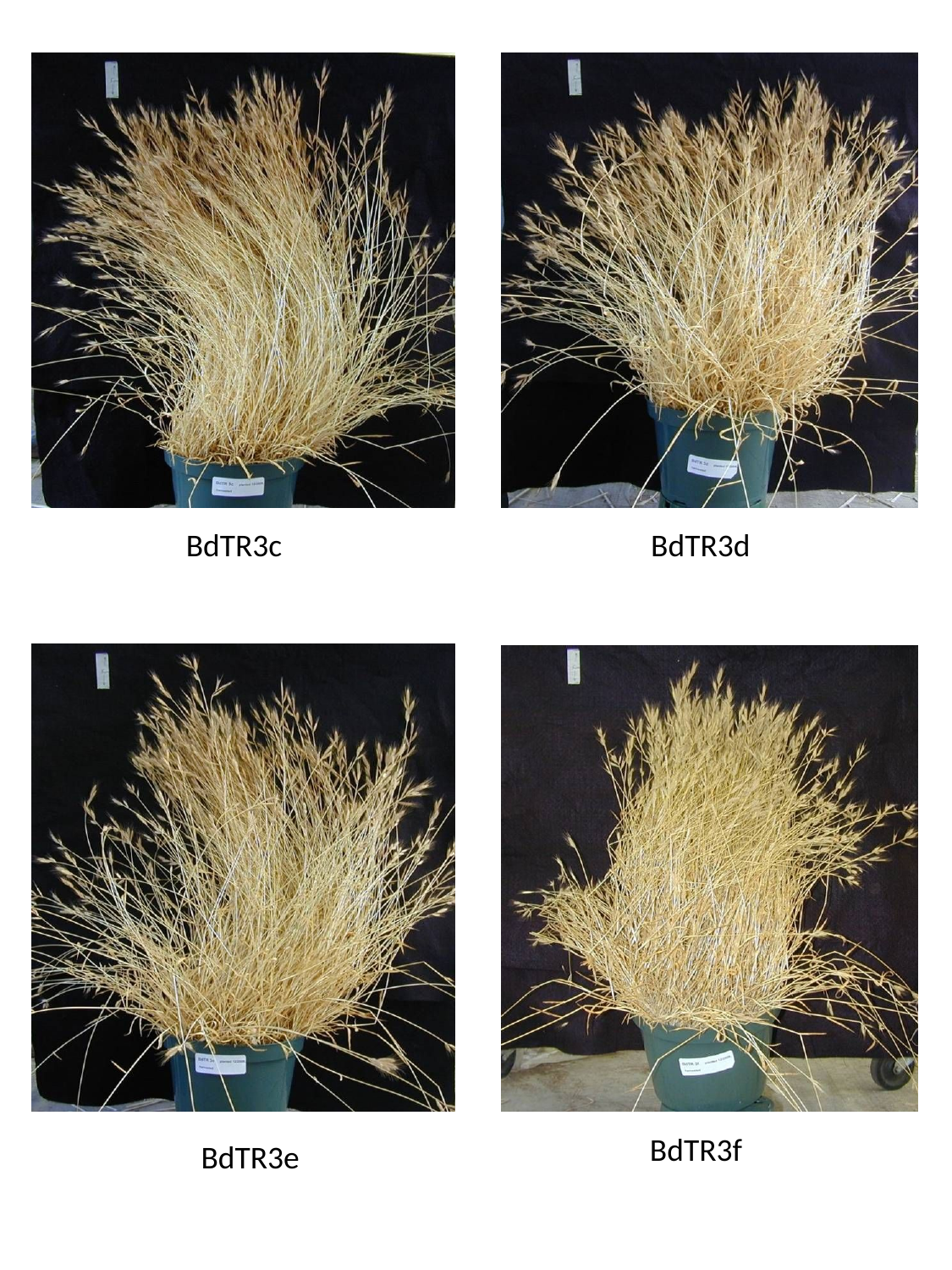

BdTR3c
BdTR3d
BdTR3f
BdTR3e

## Slide 16
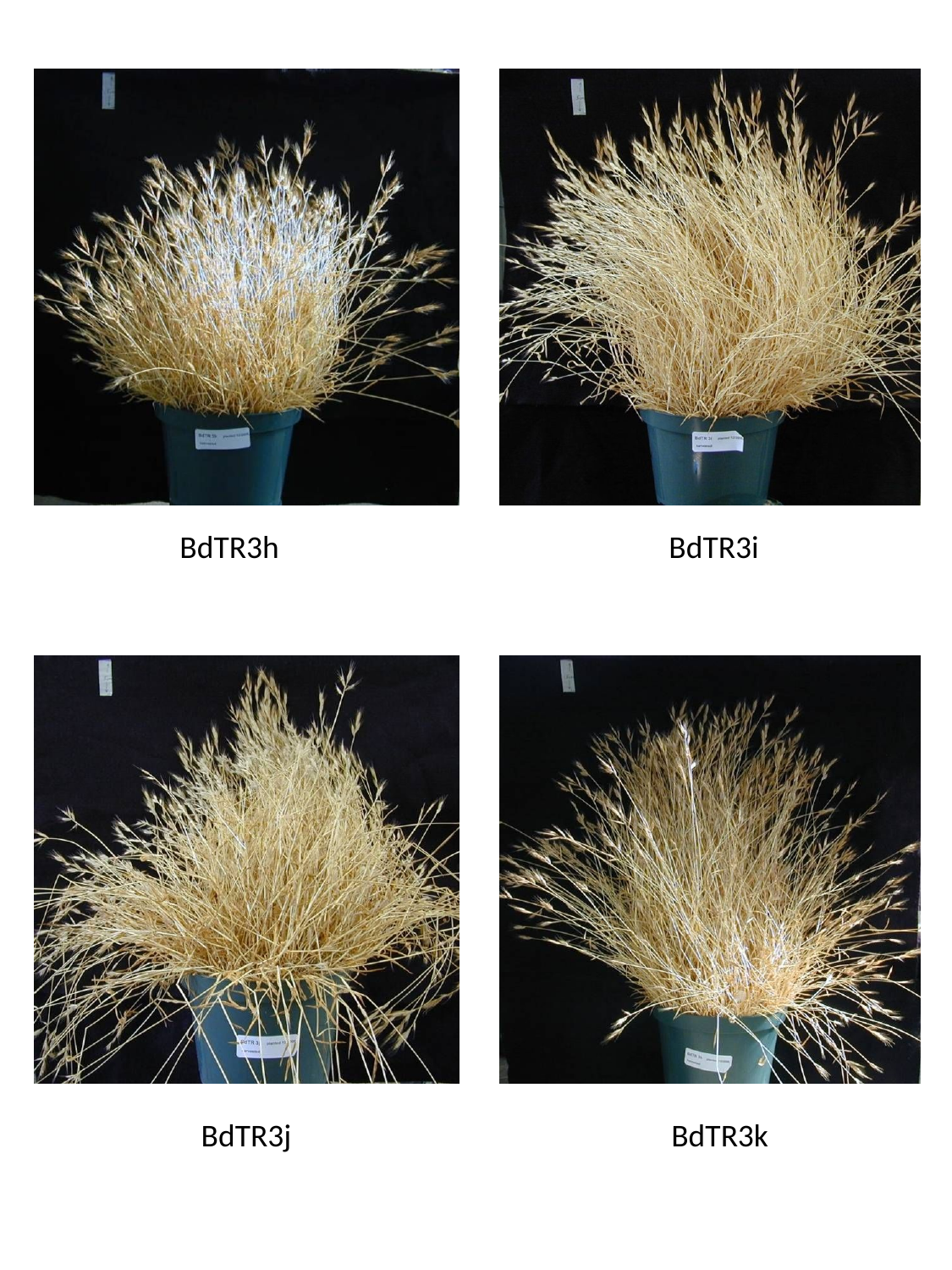

BdTR3h
BdTR3i
BdTR3j
BdTR3k

## Slide 17
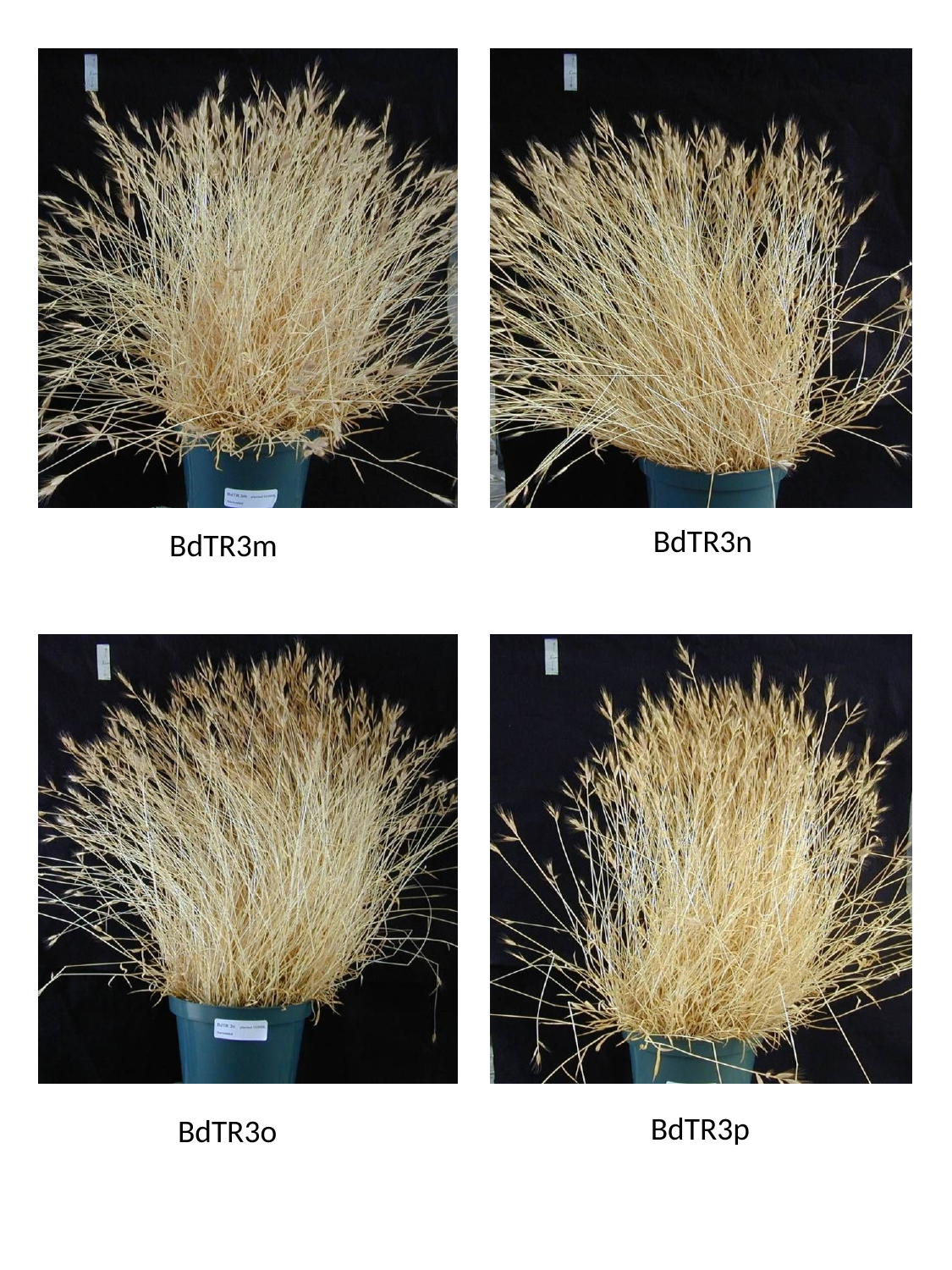

BdTR3n
BdTR3m
BdTR3p
BdTR3o

## Slide 18
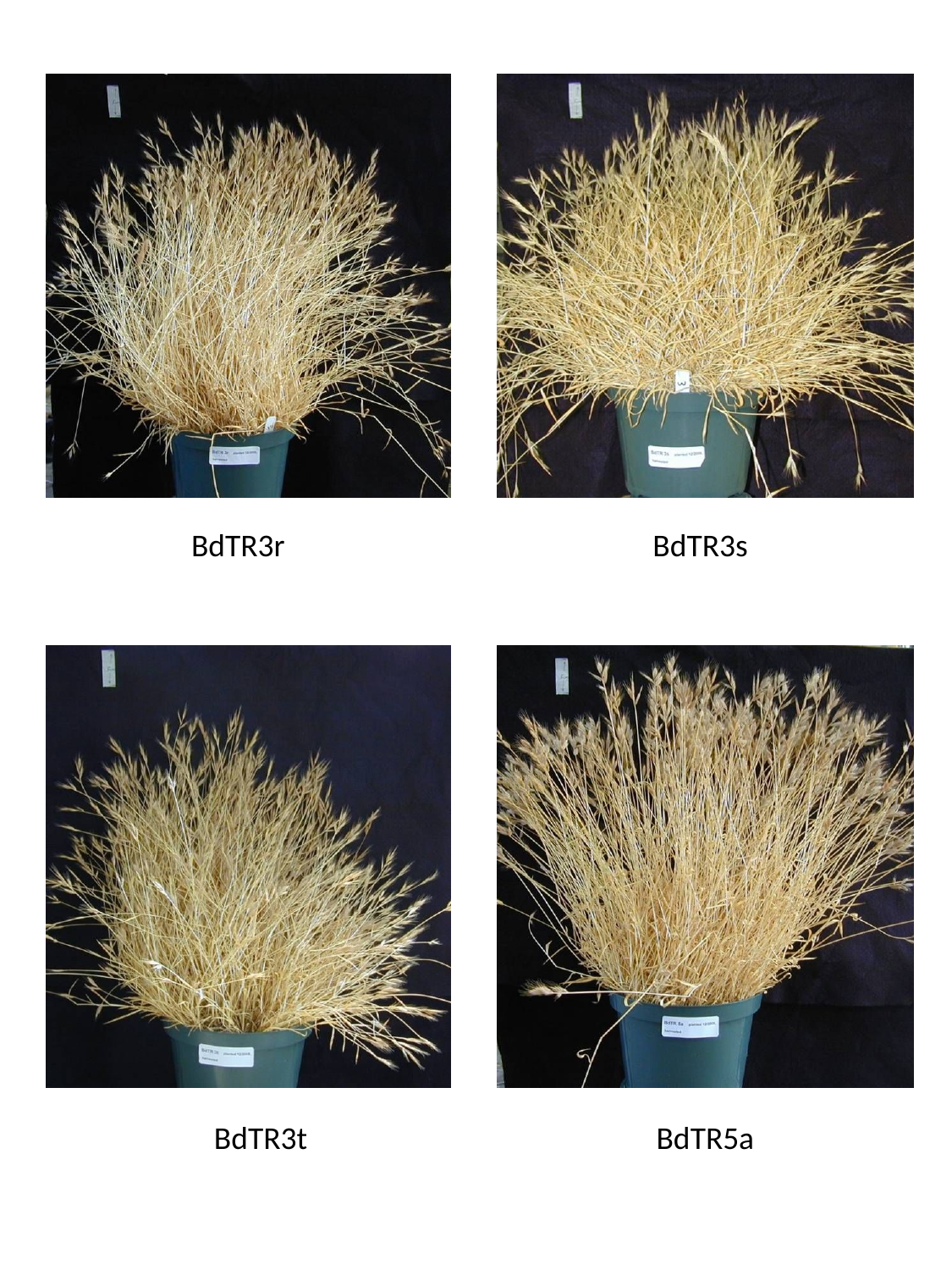

BdTR3r
BdTR3s
BdTR3t
BdTR5a

## Slide 19
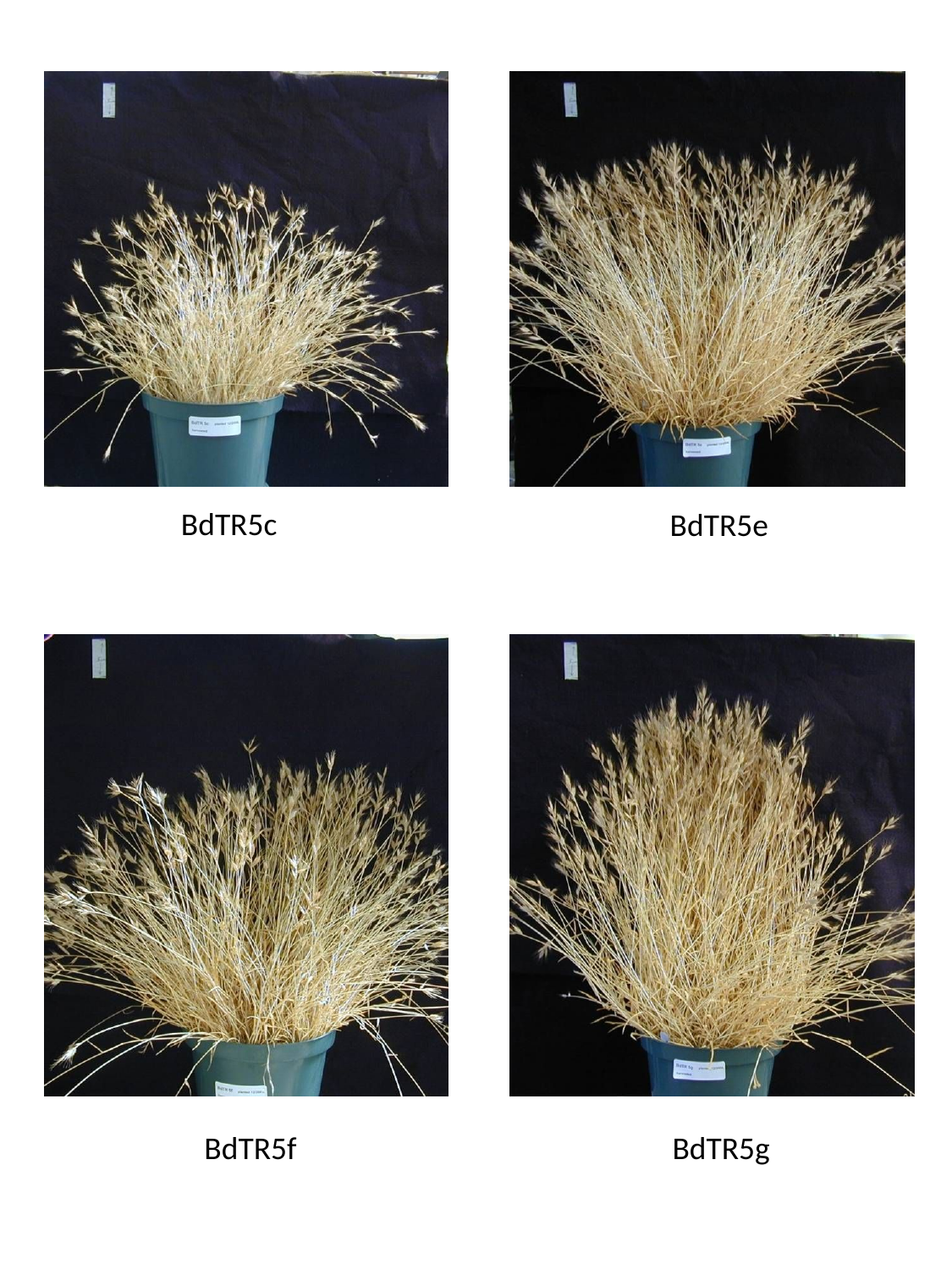

BdTR5c
BdTR5e
BdTR5f
BdTR5g

## Slide 20
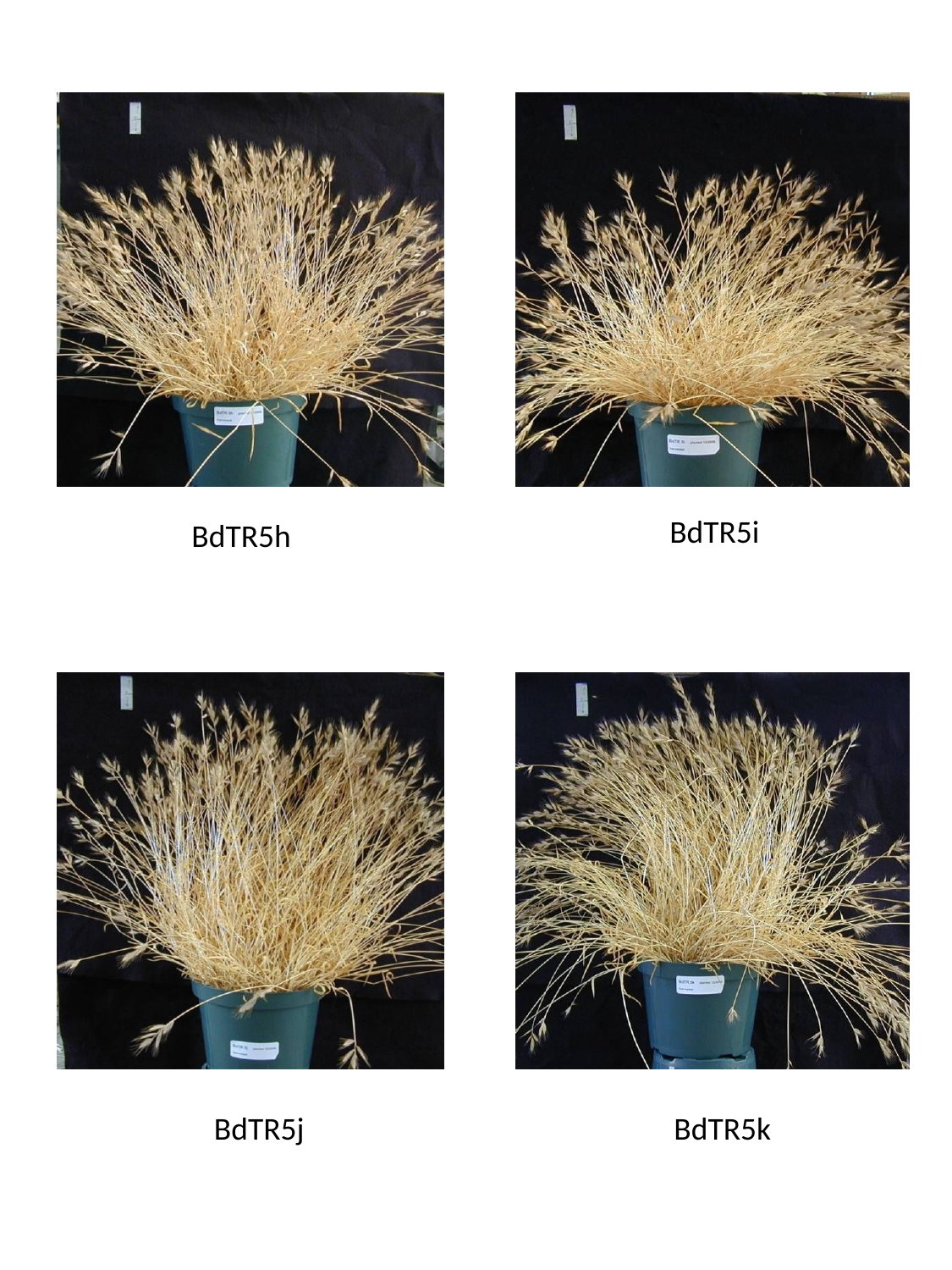

BdTR5i
BdTR5h
BdTR5j
BdTR5k

## Slide 21
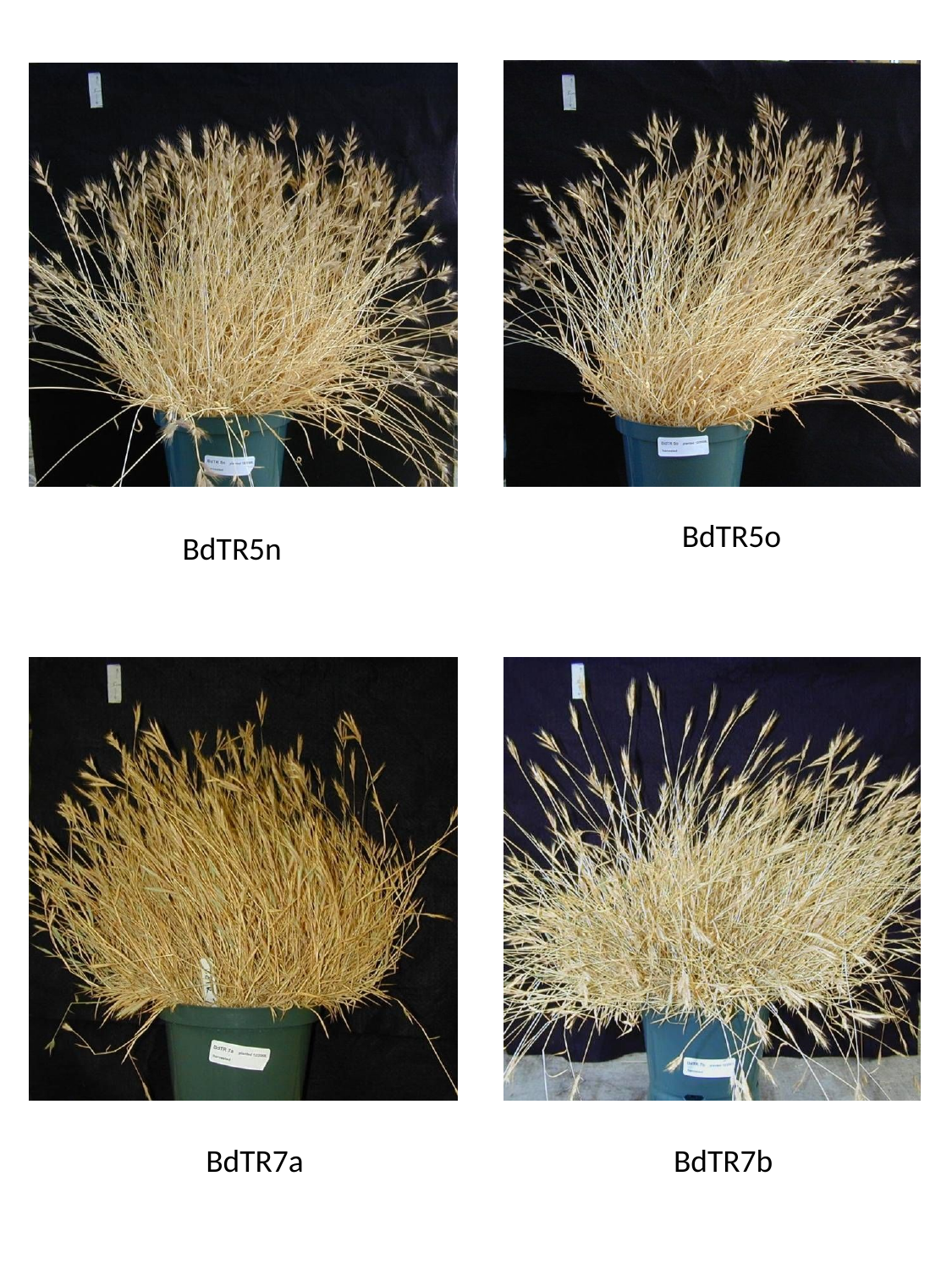

BdTR5o
BdTR5n
BdTR7a
BdTR7b

## Slide 22
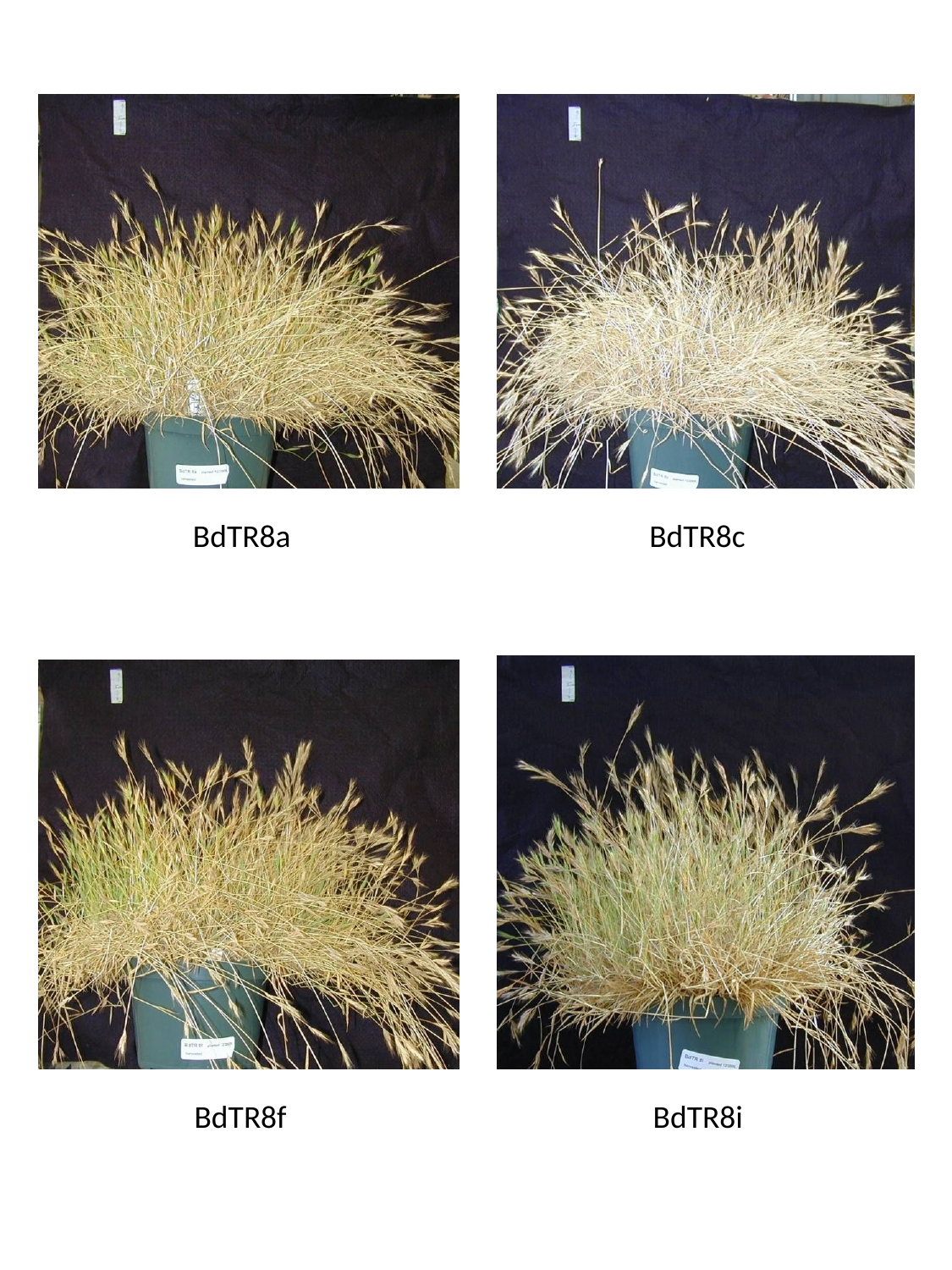

BdTR8a
BdTR8c
BdTR8i
BdTR8f

## Slide 23
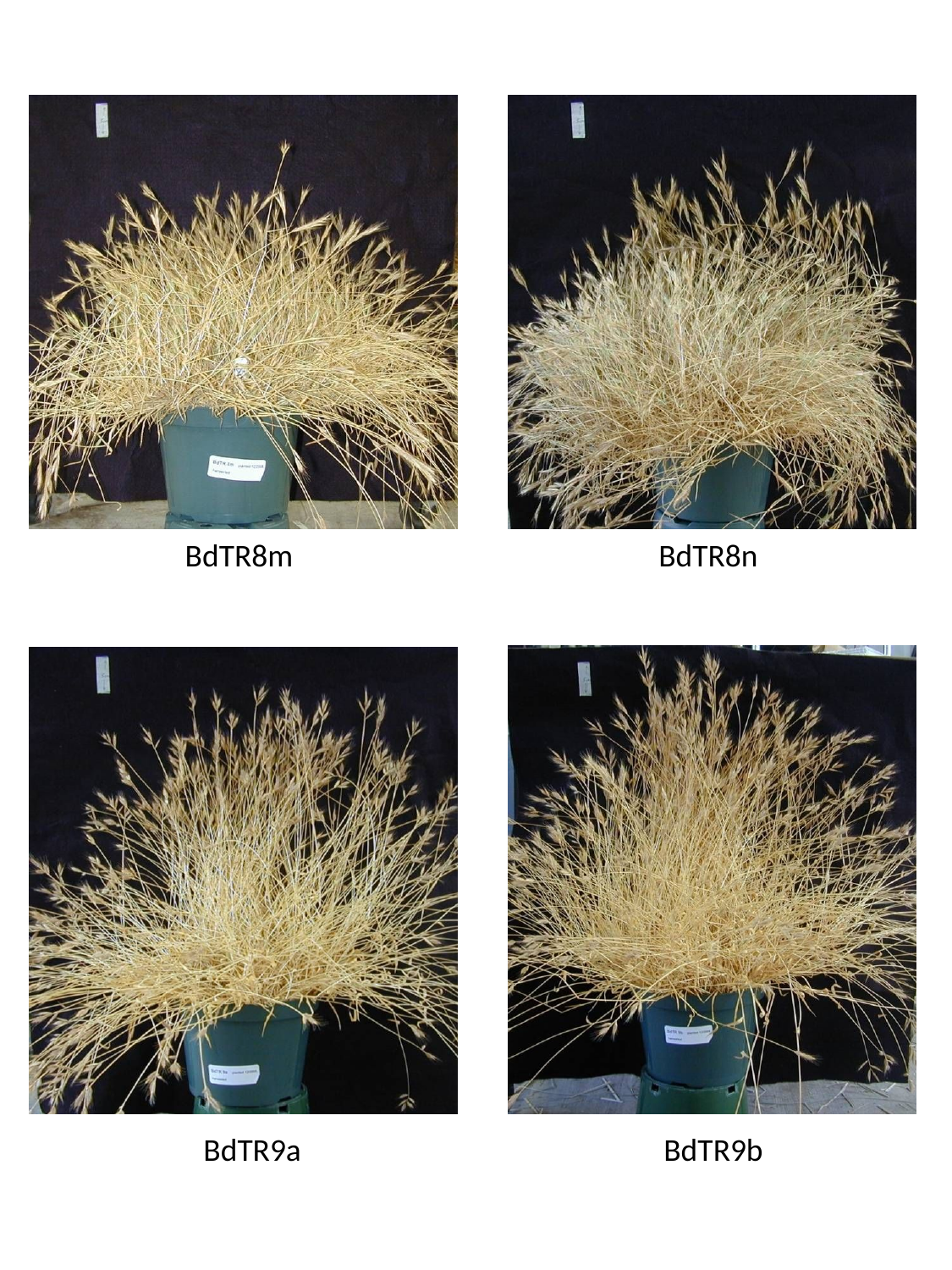

BdTR8m
BdTR8n
BdTR9a
BdTR9b

## Slide 24
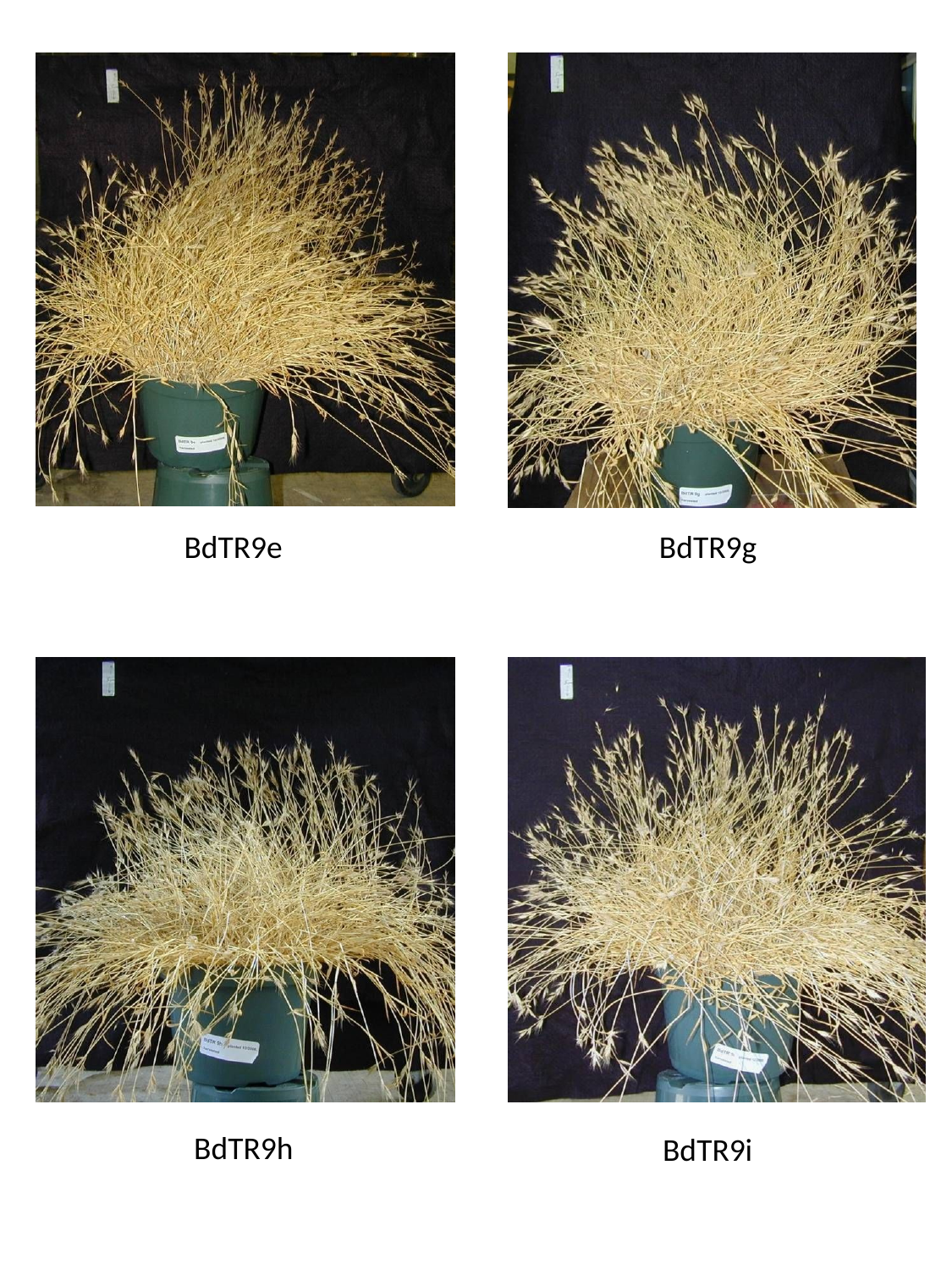

BdTR9e
BdTR9g
BdTR9h
BdTR9i

## Slide 25
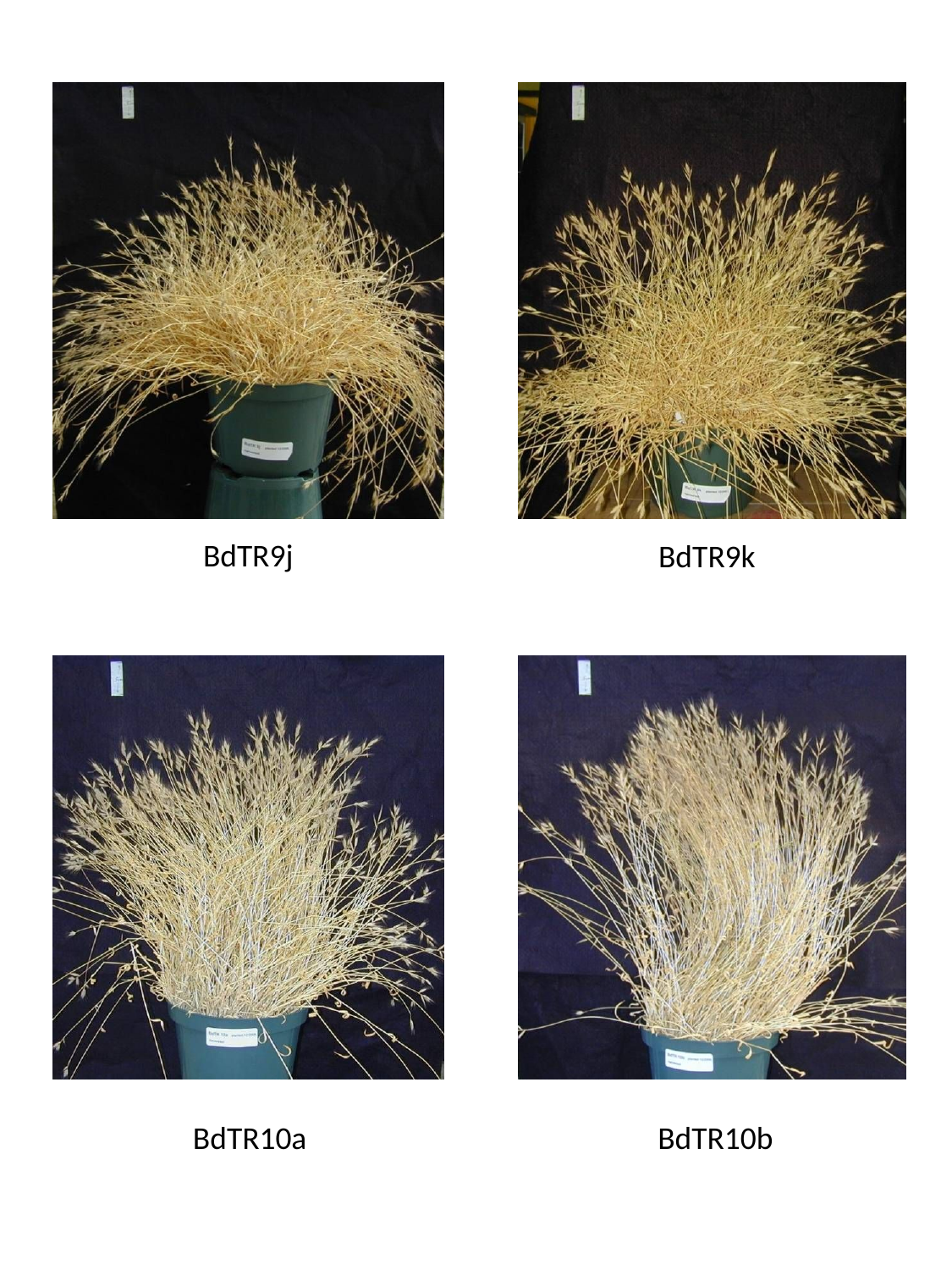

BdTR9j
BdTR9k
BdTR10a
BdTR10b

## Slide 26
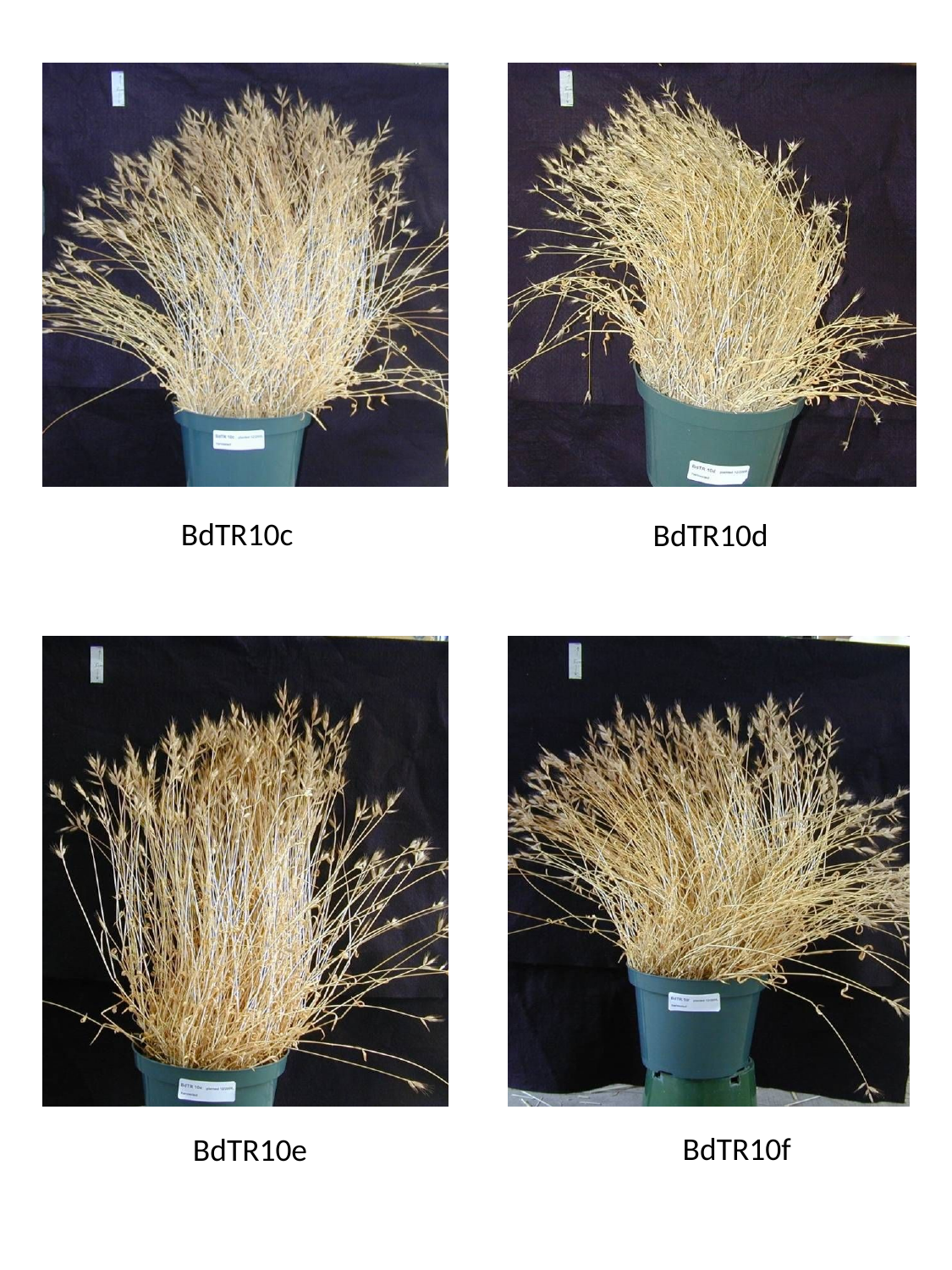

BdTR10c
BdTR10d
BdTR10f
BdTR10e

## Slide 27
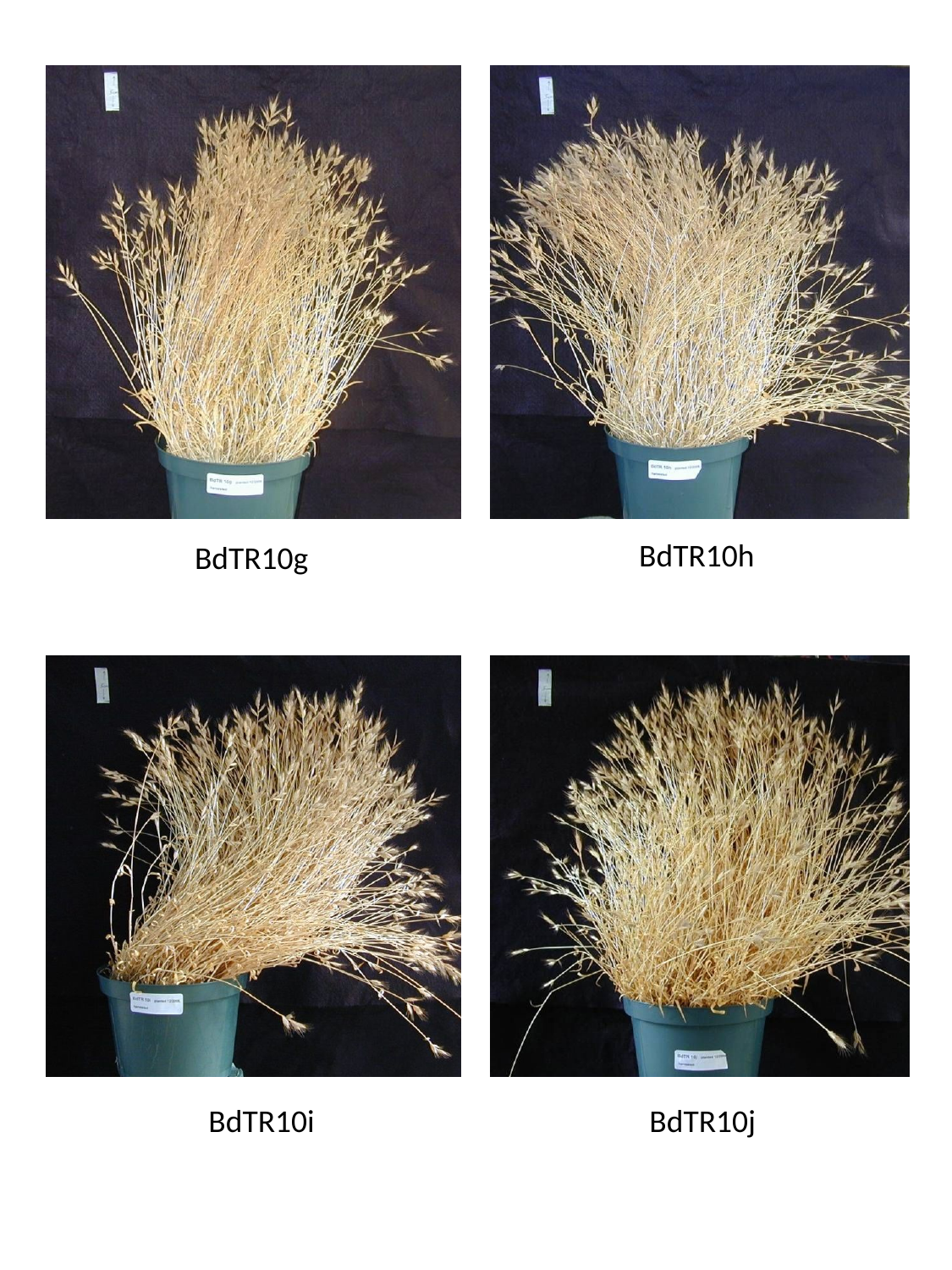

BdTR10h
BdTR10g
BdTR10i
BdTR10j

## Slide 28
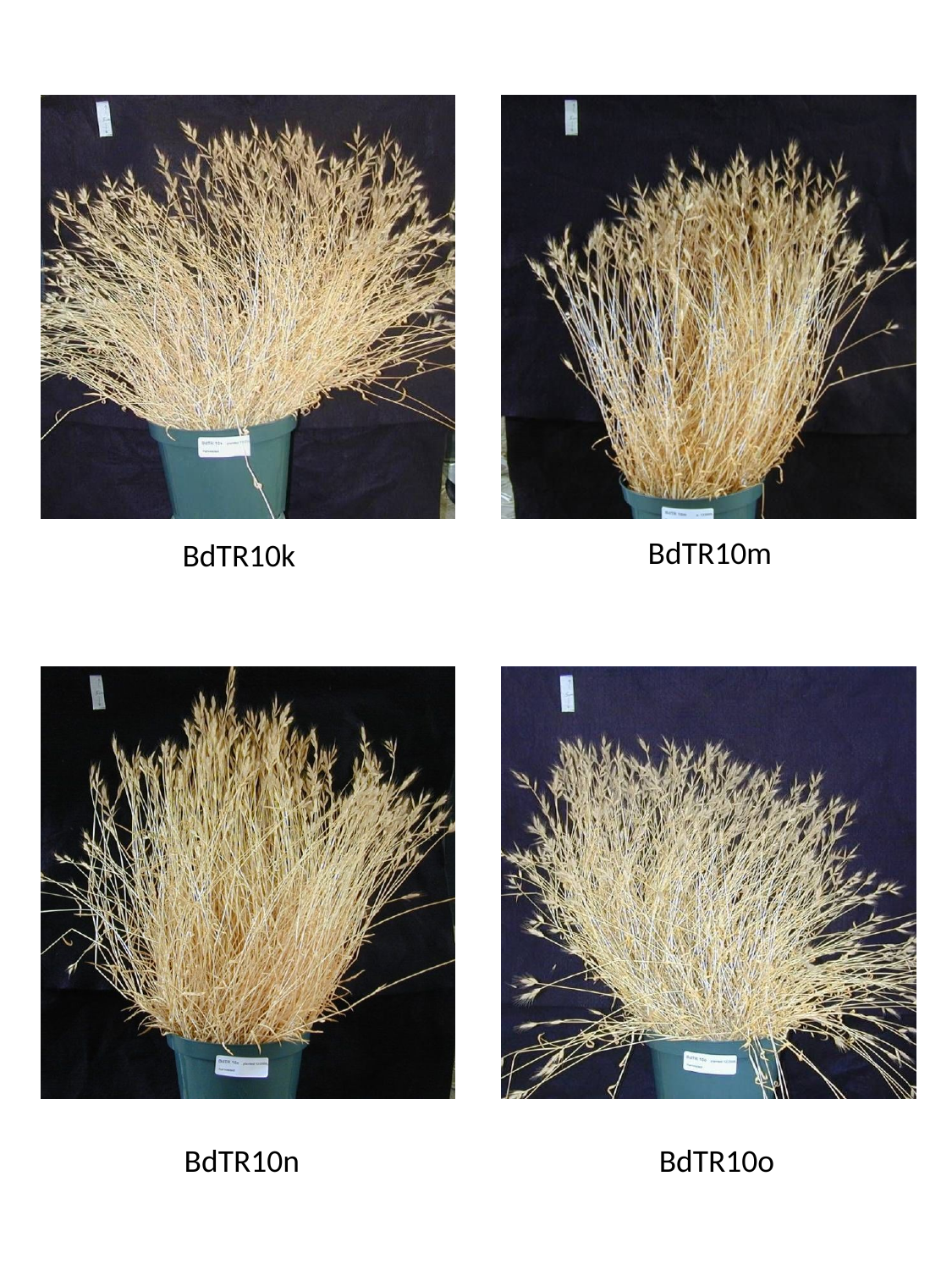

BdTR10m
BdTR10k
BdTR10n
BdTR10o

## Slide 29
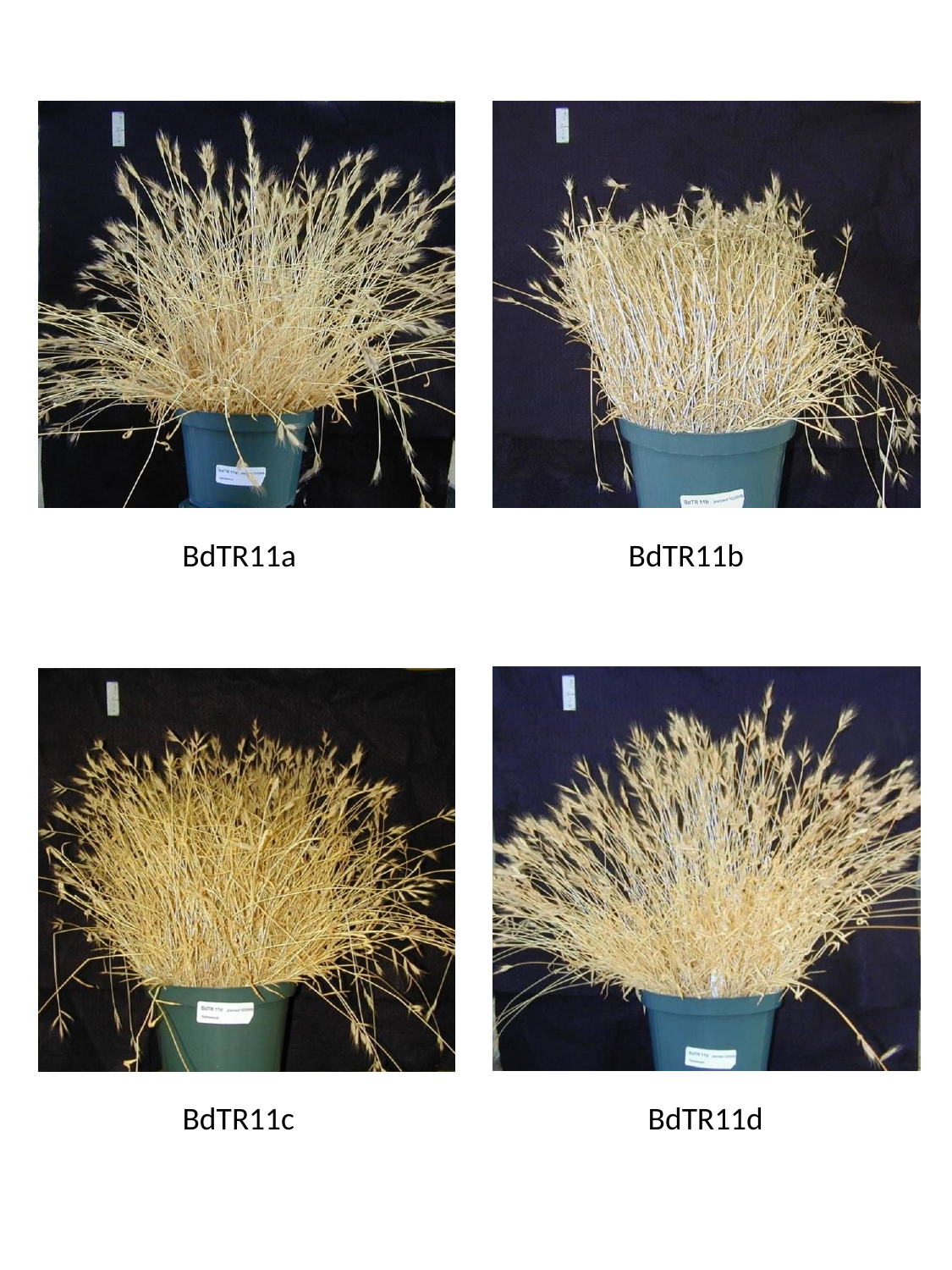

BdTR11a
BdTR11b
BdTR11c
BdTR11d

## Slide 30
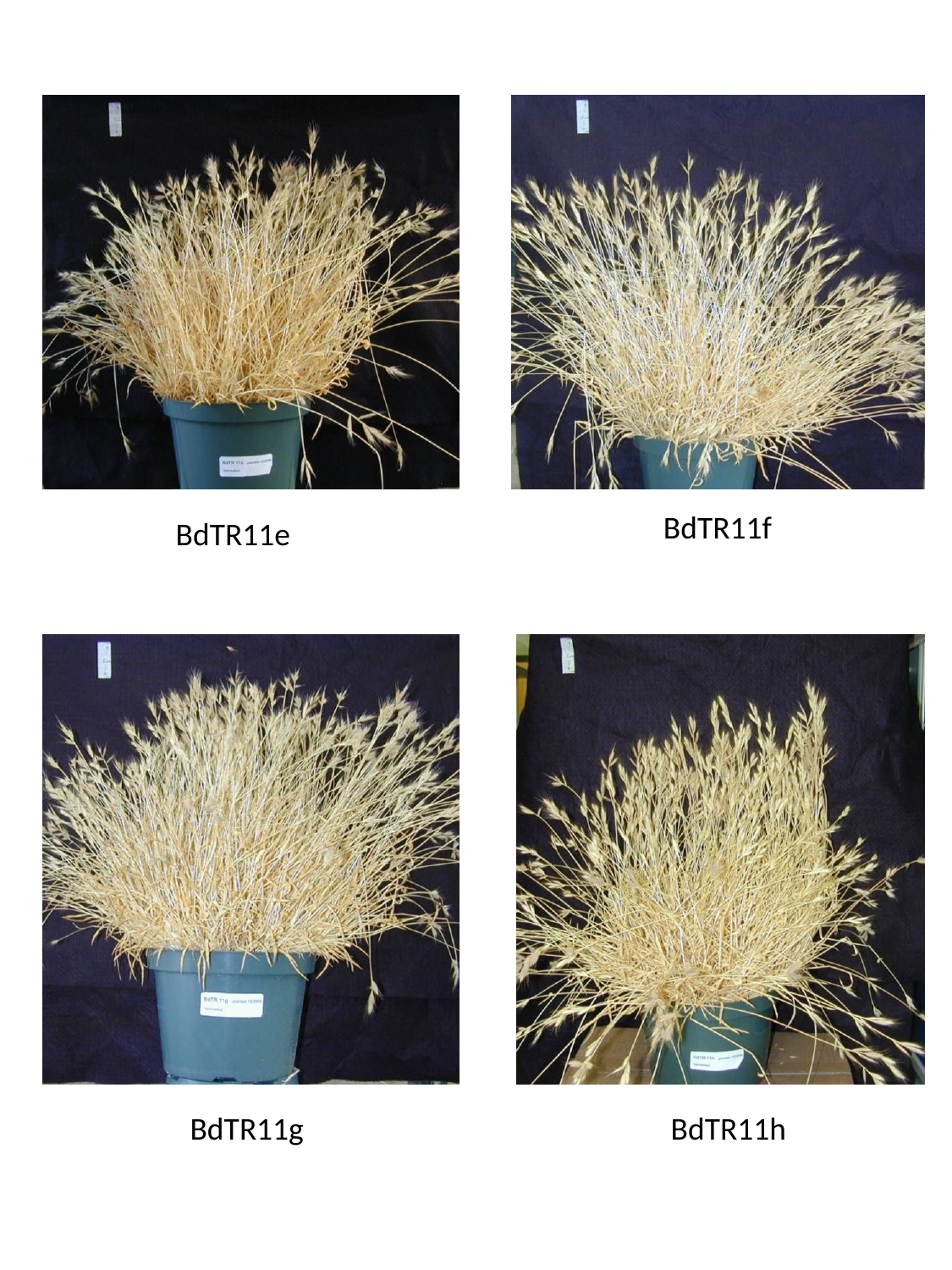

BdTR11f
BdTR11e
BdTR11g
BdTR11h

## Slide 31
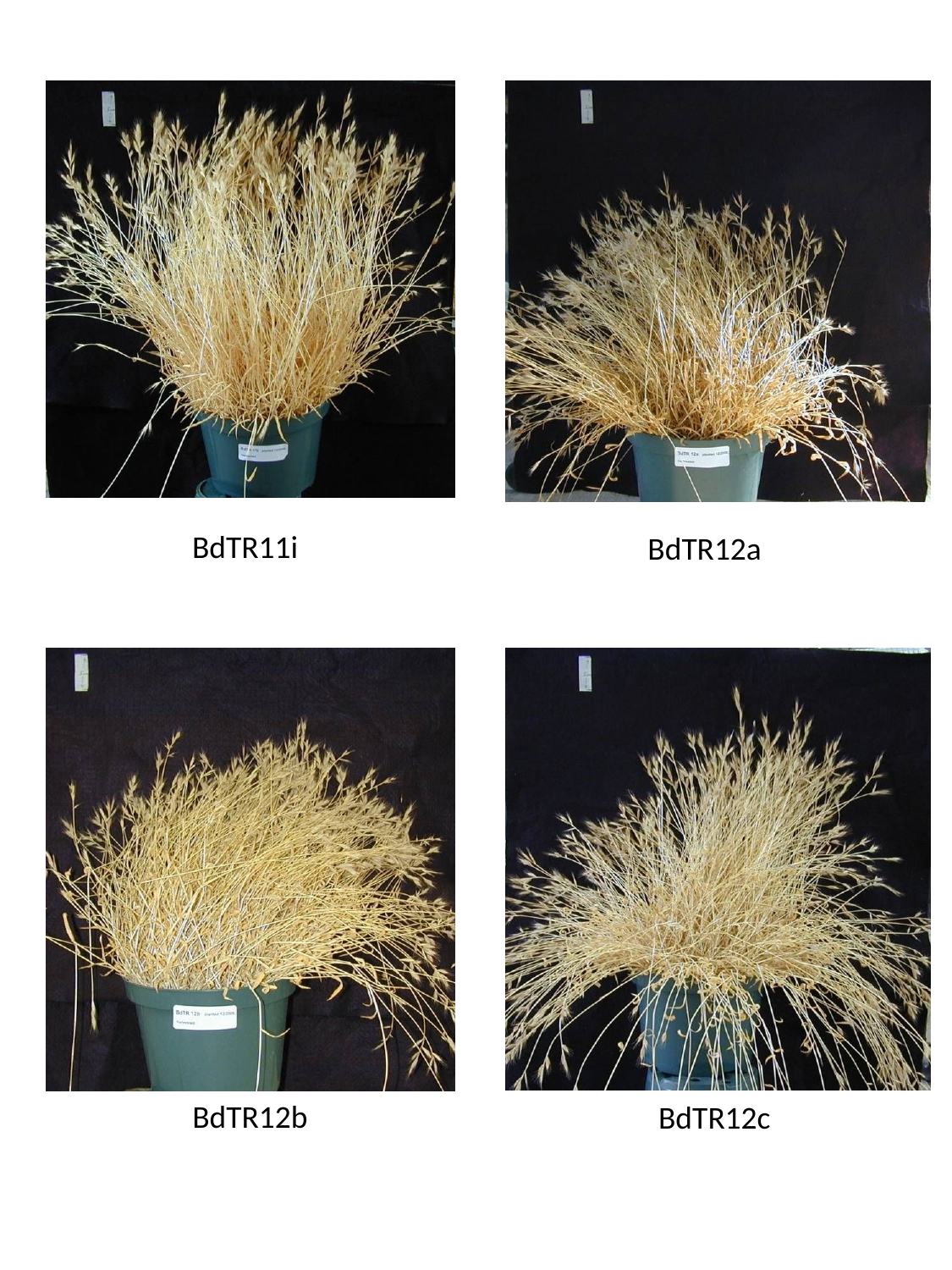

BdTR11i
BdTR12a
BdTR12b
BdTR12c

## Slide 32
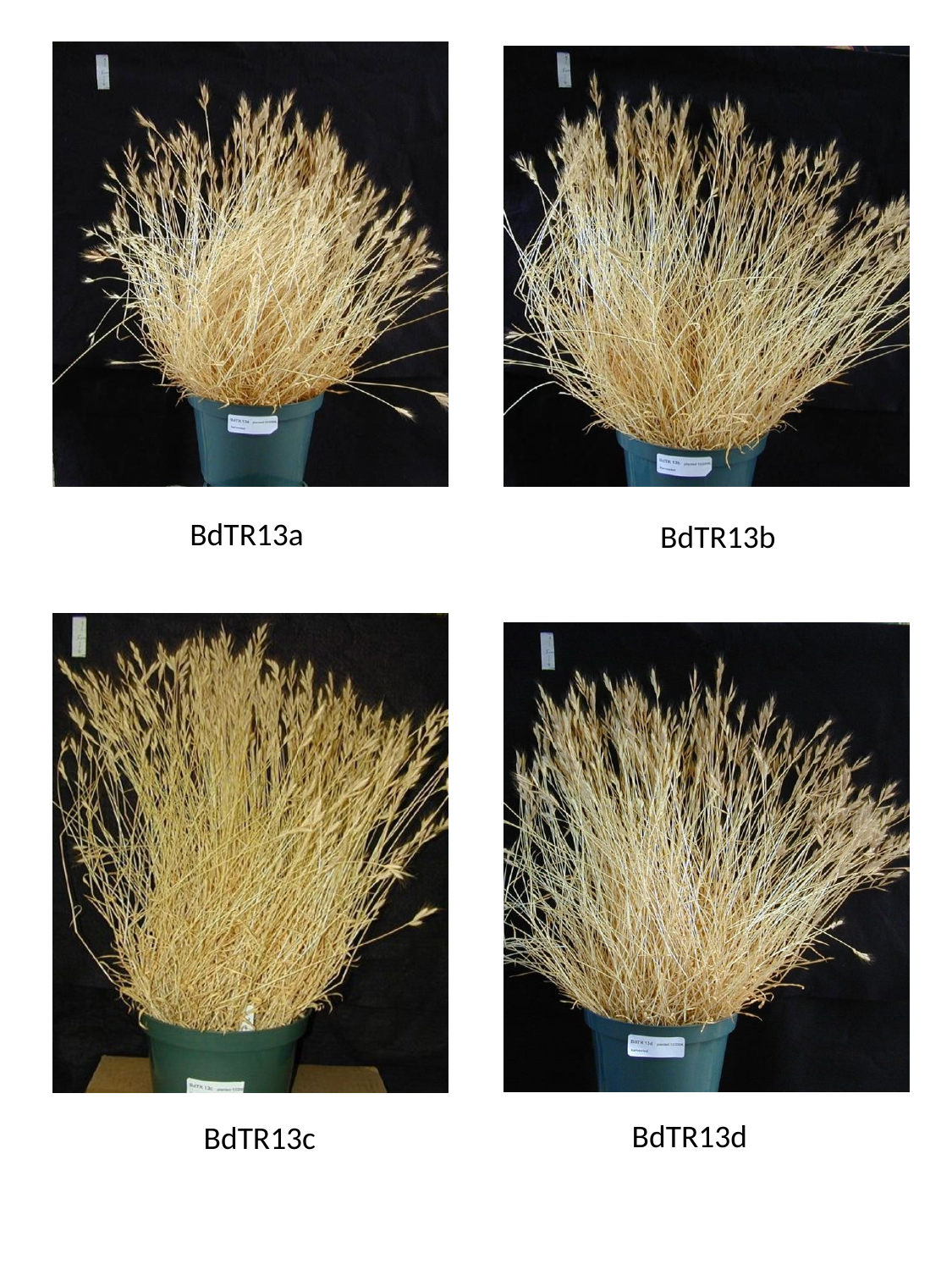

BdTR13a
BdTR13b
BdTR13d
BdTR13c

## Slide 33
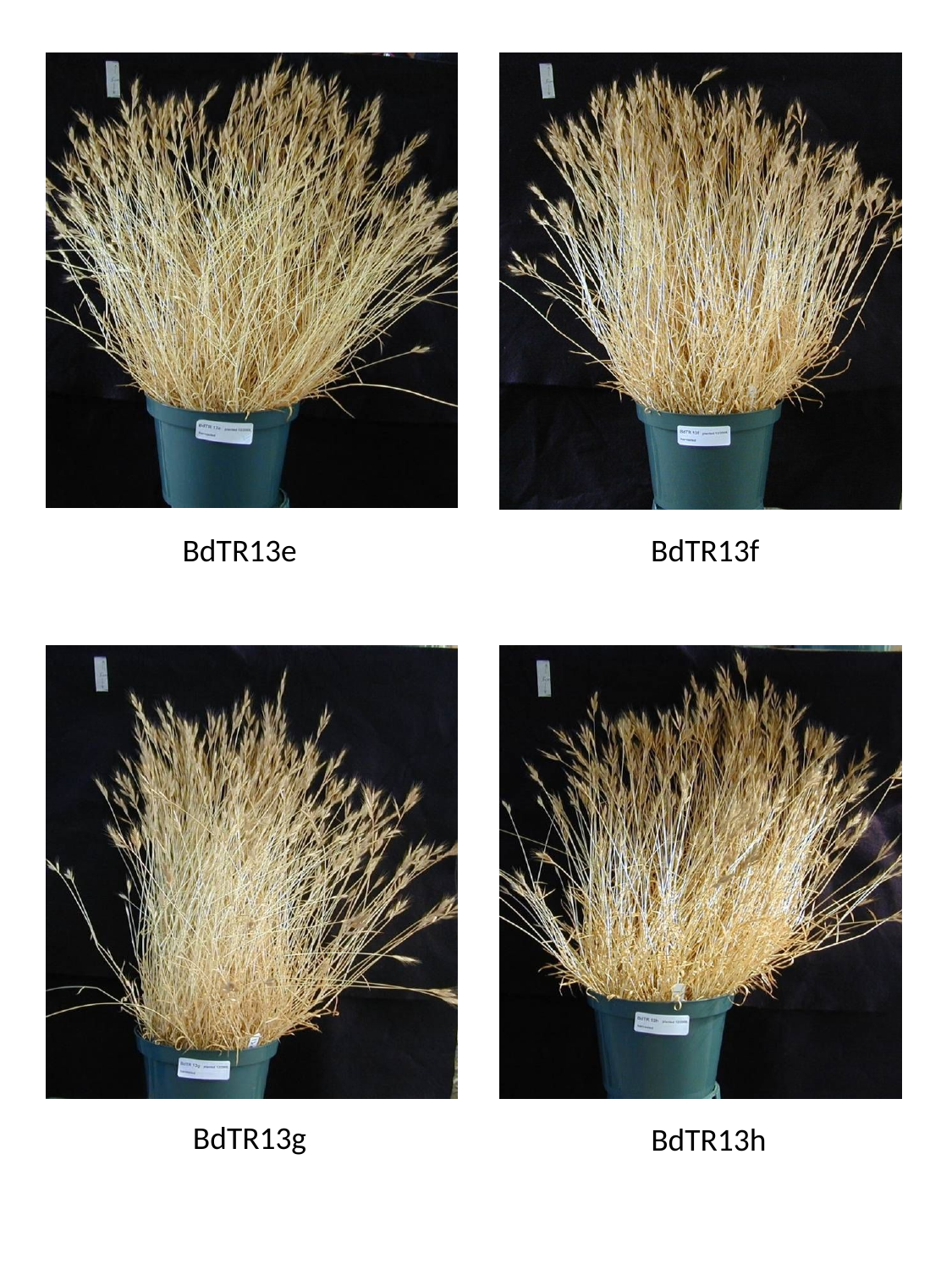

BdTR13e
BdTR13f
BdTR13g
BdTR13h

## Slide 34
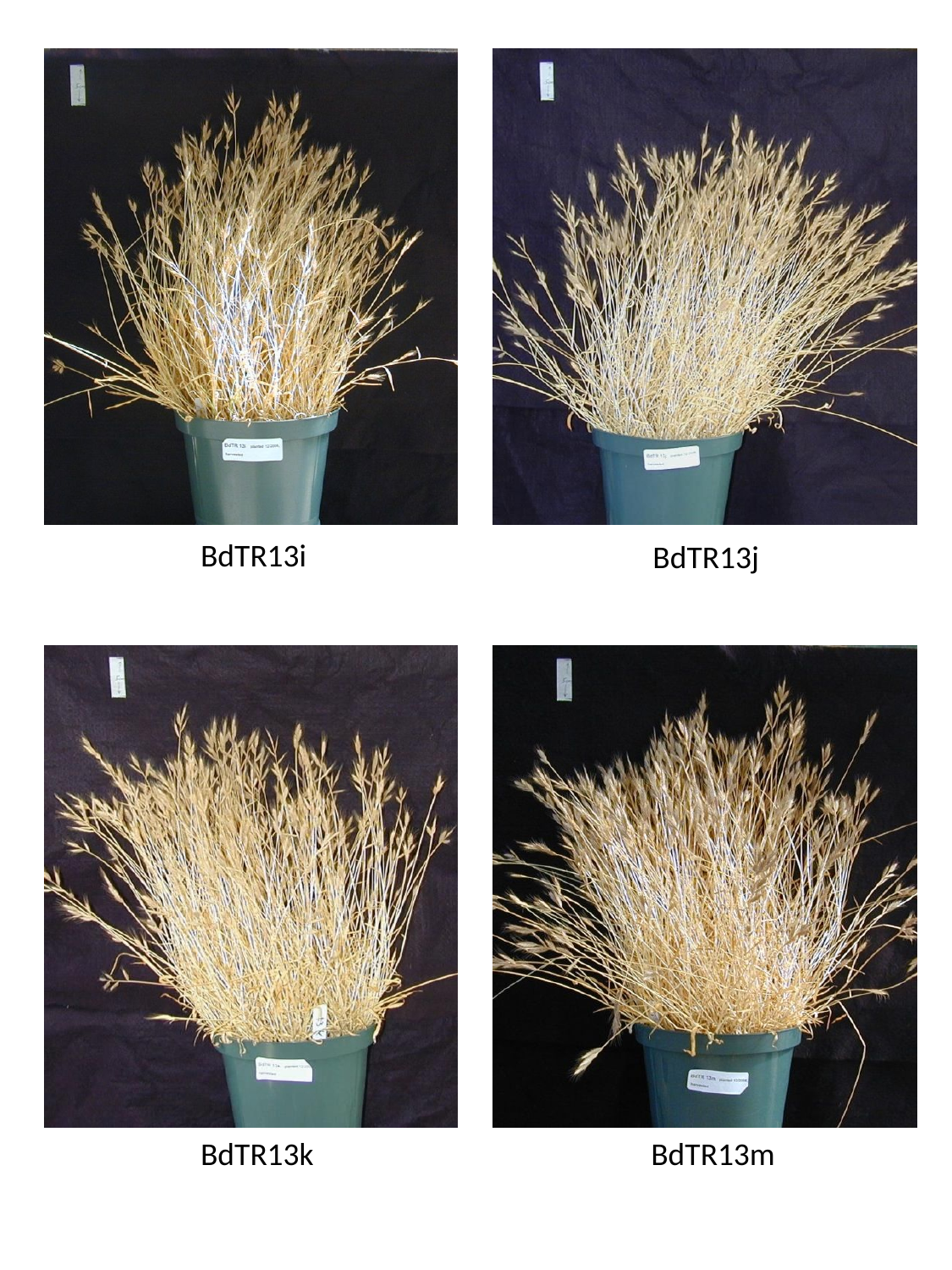

BdTR13i
BdTR13j
BdTR13k
BdTR13m

## Slide 35
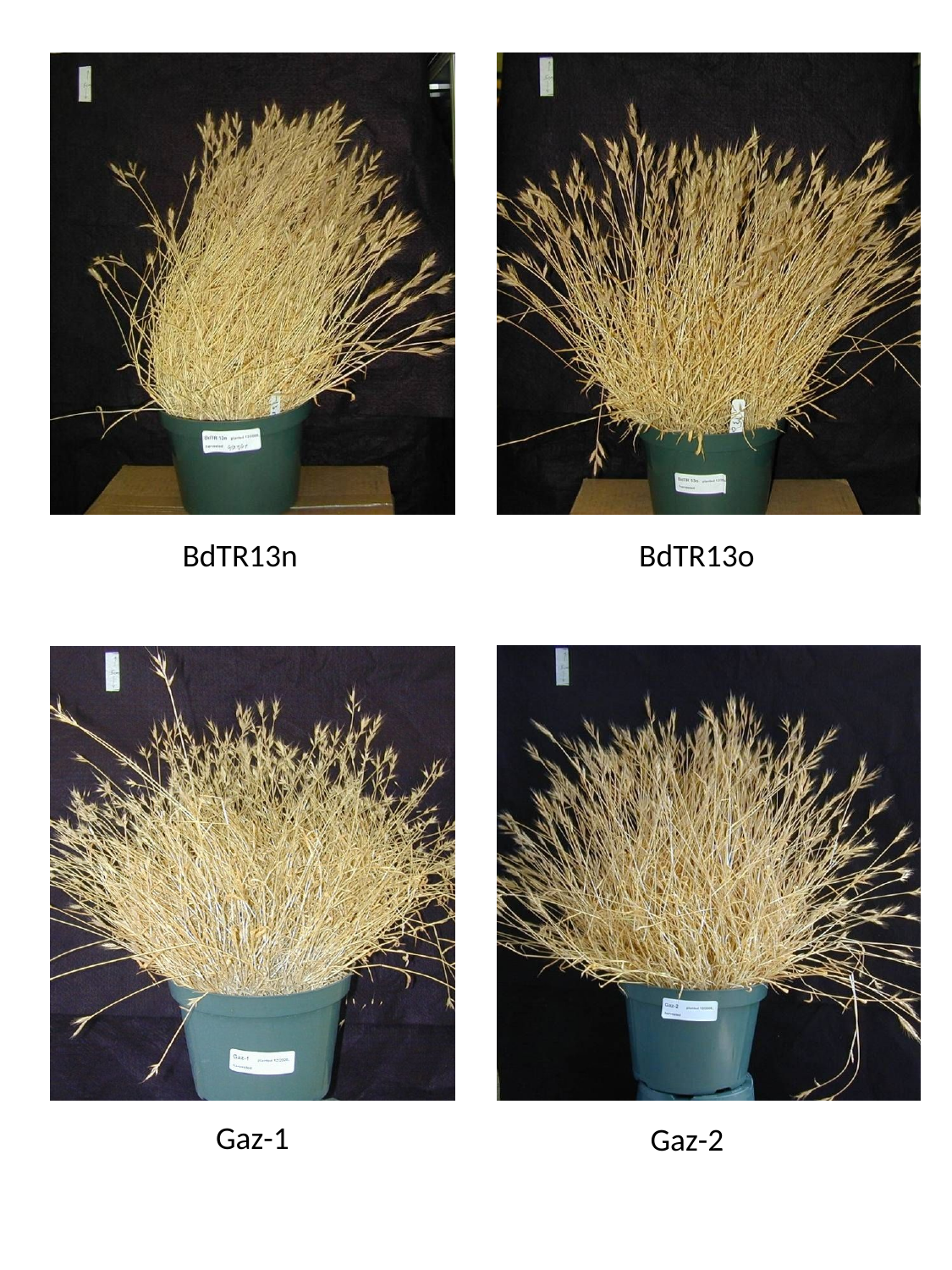

BdTR13n
BdTR13o
Gaz-1
Gaz-2

## Slide 36
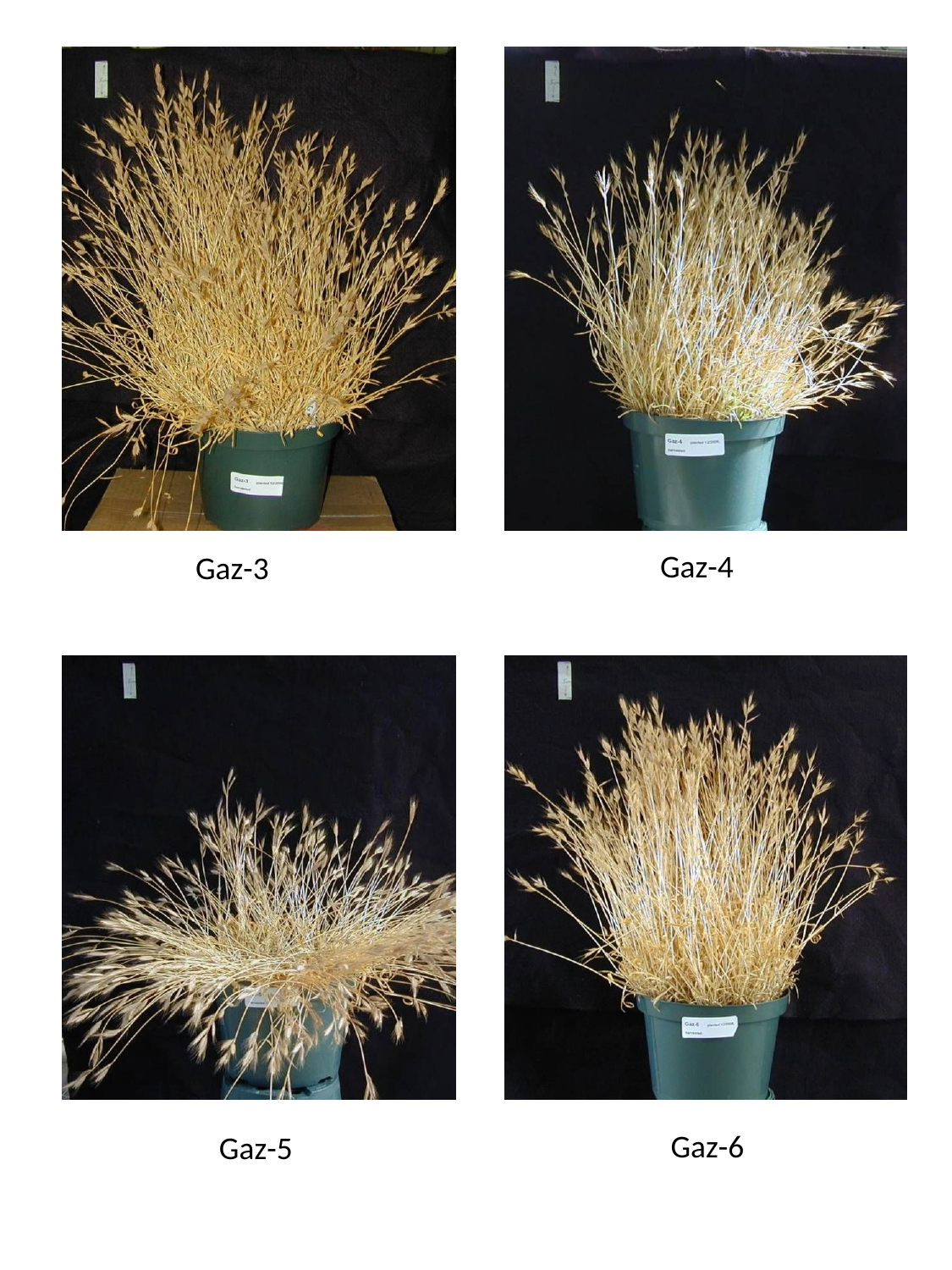

Gaz-4
Gaz-3
Gaz-6
Gaz-5

## Slide 37
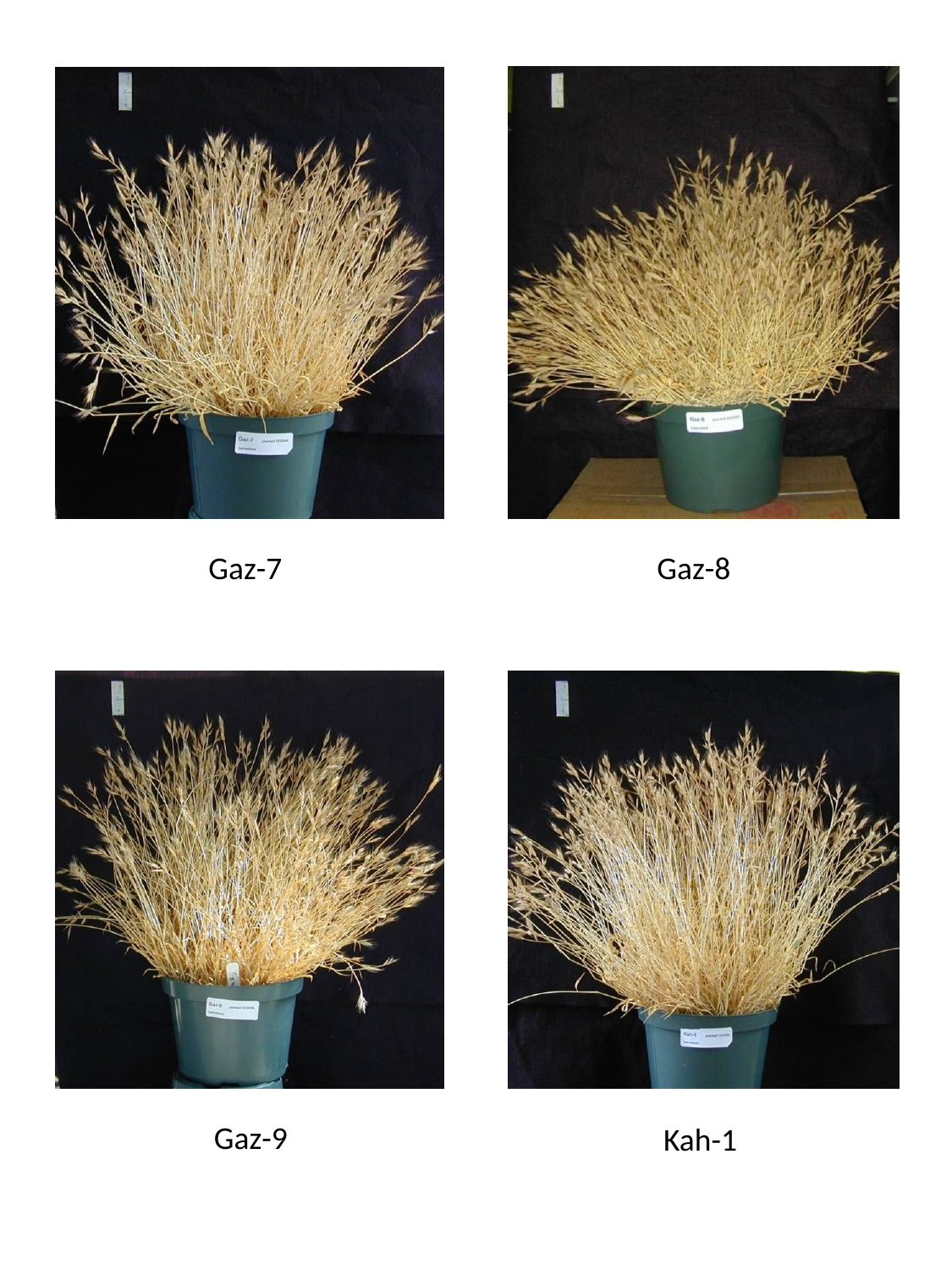

Gaz-7
Gaz-8
Gaz-9
Kah-1

## Slide 38
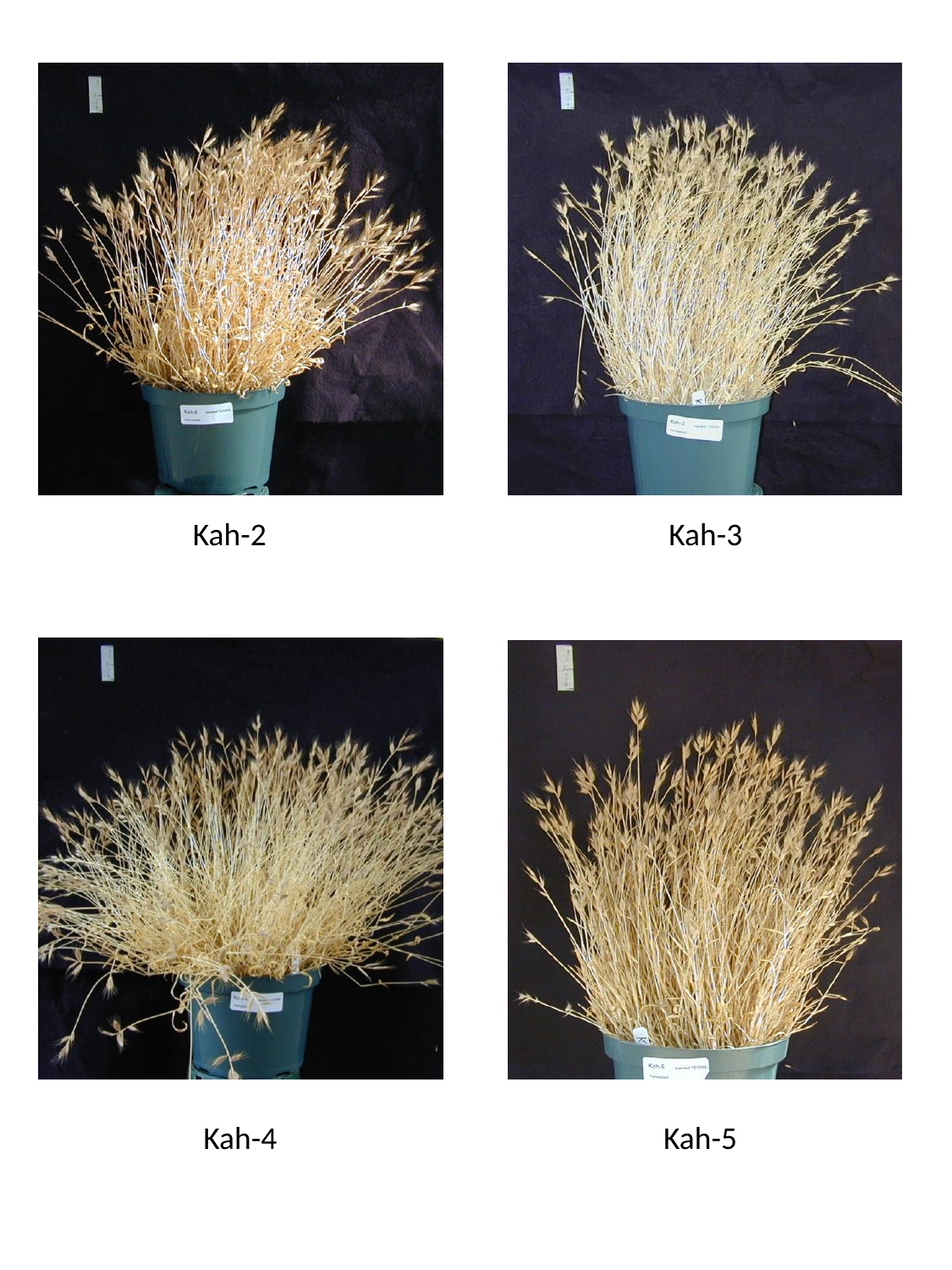

Kah-2
Kah-3
Kah-4
Kah-5

## Slide 39
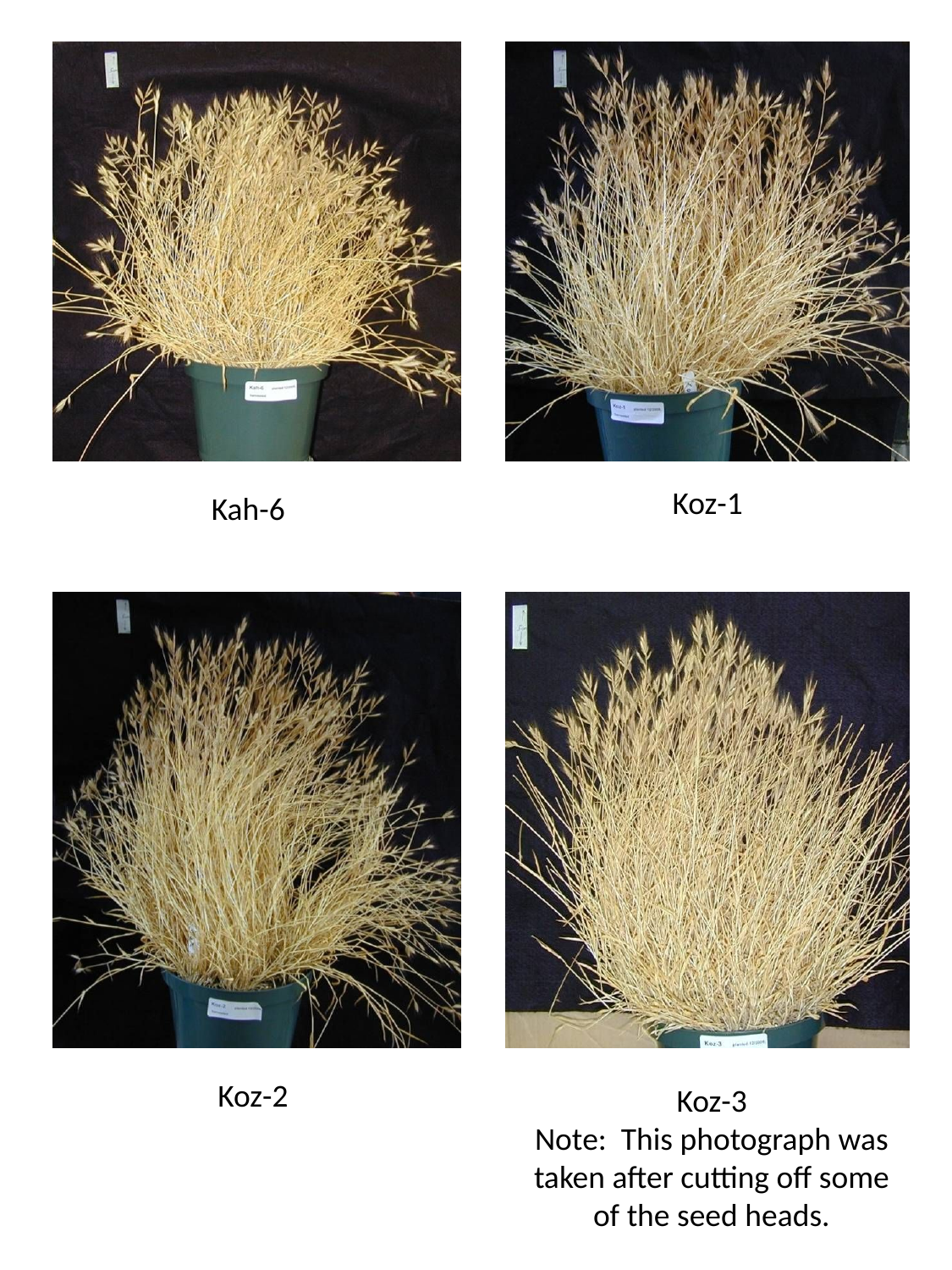

Koz-1
Kah-6
Koz-2
Koz-3
Note: This photograph was taken after cutting off some of the seed heads.

## Slide 40
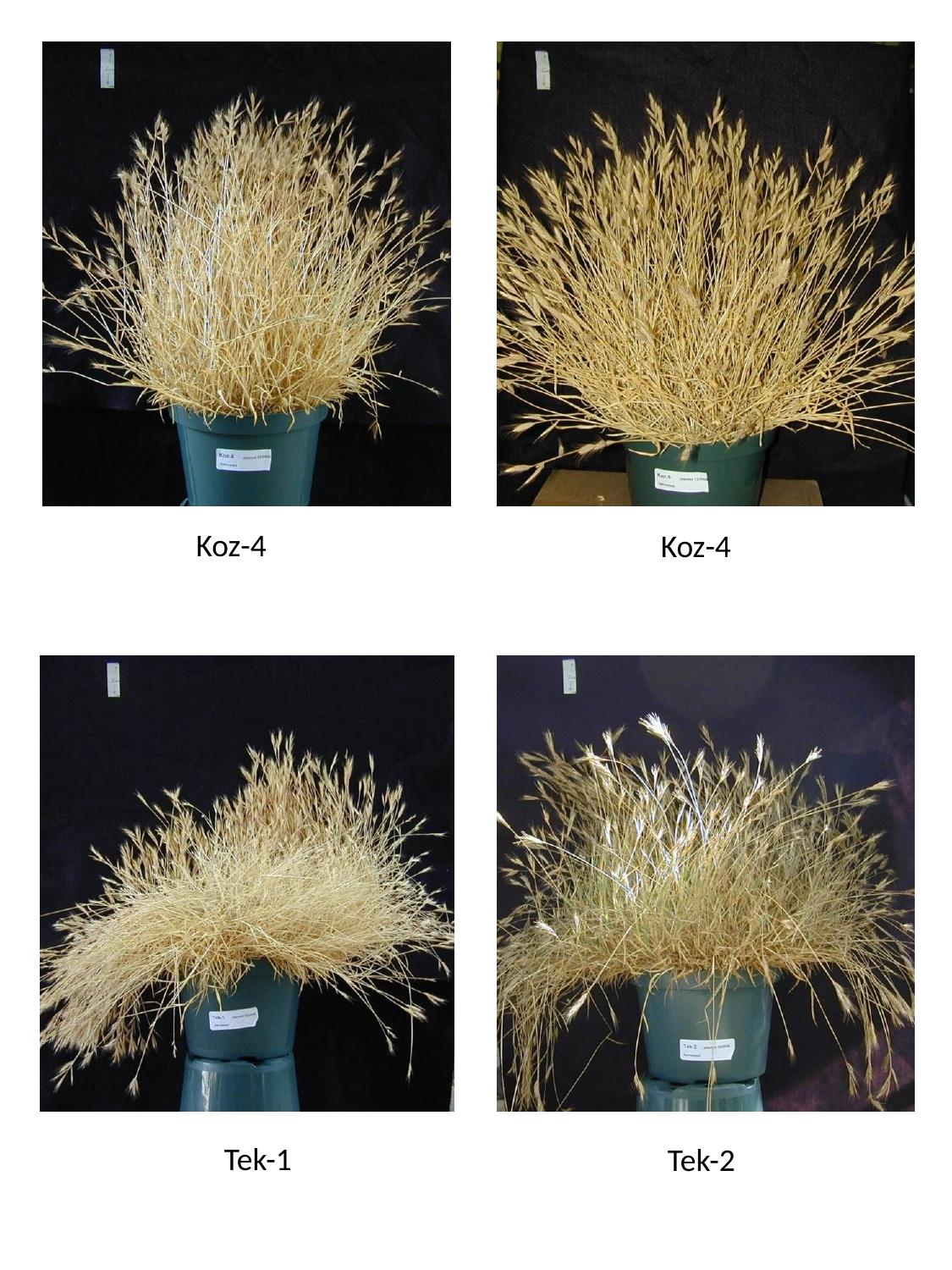

Koz-4
Koz-4
Tek-1
Tek-2

## Slide 41
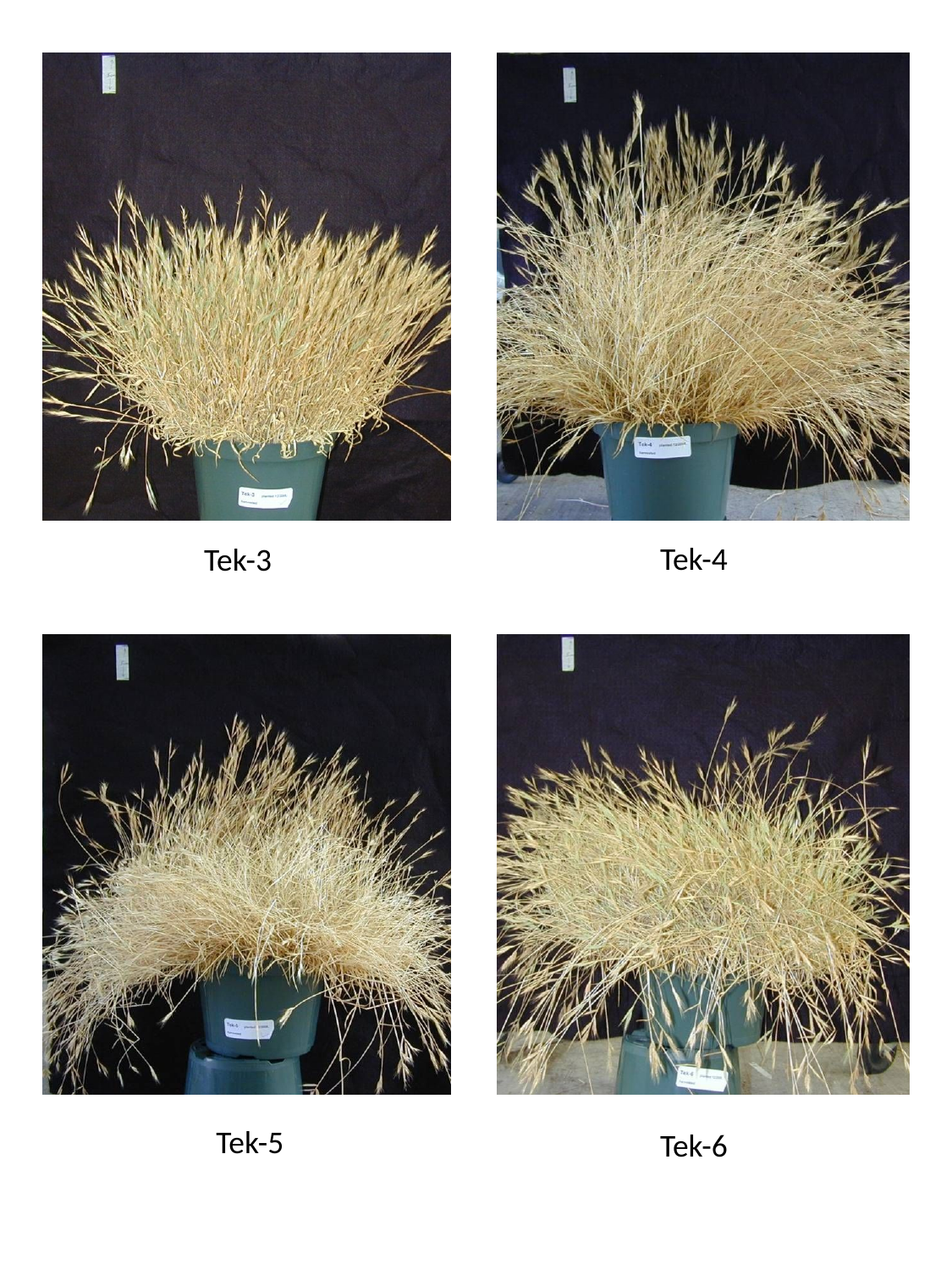

Tek-4
Tek-3
Tek-5
Tek-6

## Slide 42
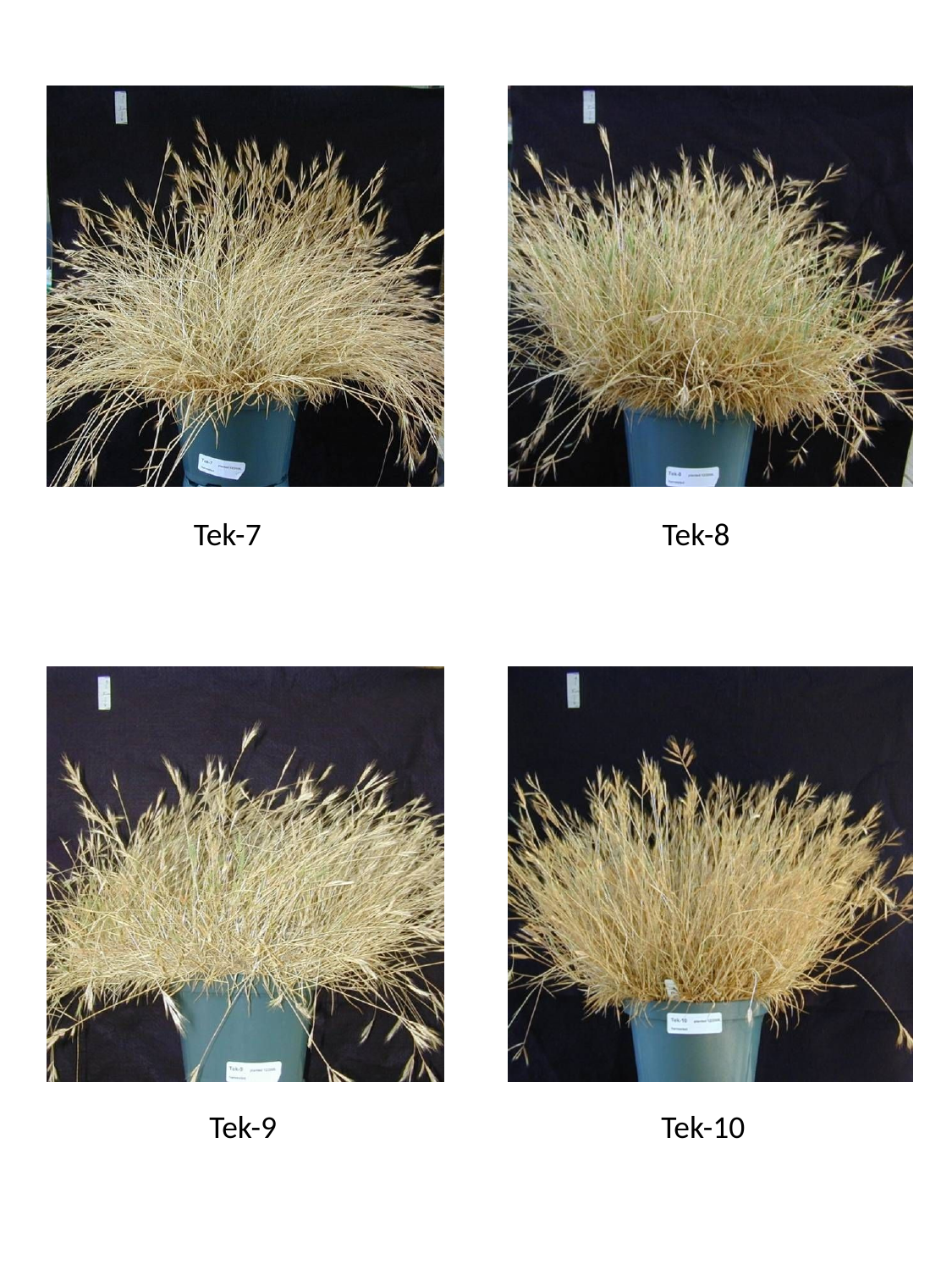

Tek-7
Tek-8
Tek-9
Tek-10

## Slide 43
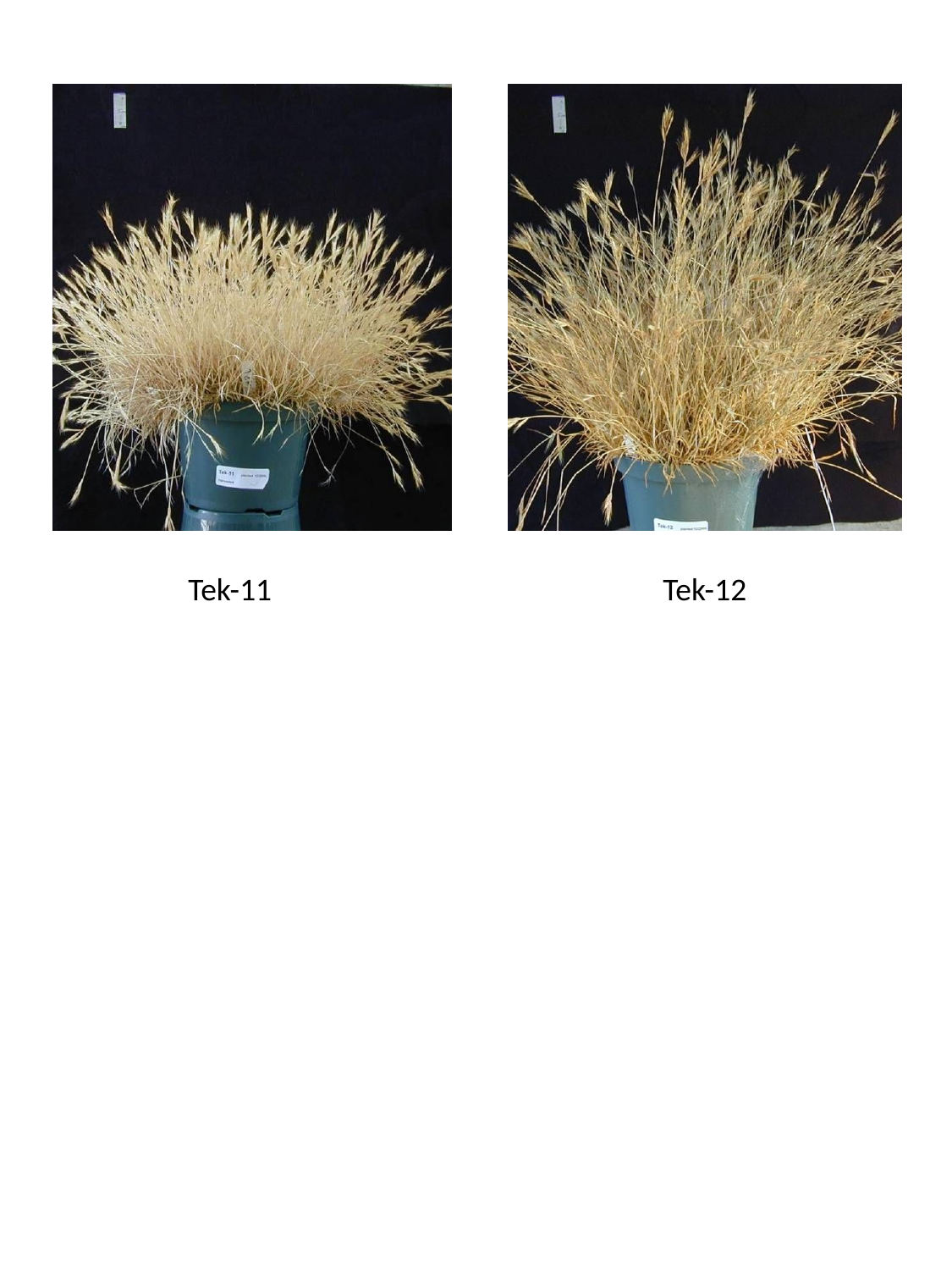

Tek-11
Tek-12
